# Challenges and progress in oxygen evolution reaction catalyst development for seawater electrolysis for hydrogen production

**DOI:** 10.1039/d3ra08648h

**Published:** 2024-02-20

**Authors:** Jack Corbin, Mikey Jones, Cheng Lyu, Adeline Loh, Zhenyu Zhang, Yanqui Zhu, Xiaohong Li

**Affiliations:** a Renewable Energy Group, Department of Engineering, Faculty of Environment, Science and Economy, University of Exeter, Penryn Campus Cornwall TR10 9FE UK; b Department of Engineering, Faculty of Environment, Science and Economy, University of Exeter, Streatham Campus Exeter EX4 4PY UK X.Li@exeter.ac.uk

## Abstract

Production of green hydrogen on a large scale can negatively impact freshwater resources. Therefore, using seawater as an electrolyte in electrolysis is a desirable alternative to reduce costs and freshwater reliance. However, there are limitations to this approach, primarily due to the catalyst involved in the oxygen evolution reaction (OER). In seawater, the OER features sluggish kinetics and complicated chemical reactions that compete. This review first introduces the benefits and challenges of direct seawater electrolysis and then summarises recent research into cost-effective and durable OER electrocatalysts. Different modification methods for nickel-based electrocatalysts are thoroughly reviewed, and promising electrocatalysts that the authors believe deserve further exploration have been highlighted.

## Abstract 2

Modification techniques to oxygen evolution reaction (OER) electrocatalysts for direct seawater electrolysis (DSWE) to mitigate chloride corrosion from seawater.

## Graphical abstract

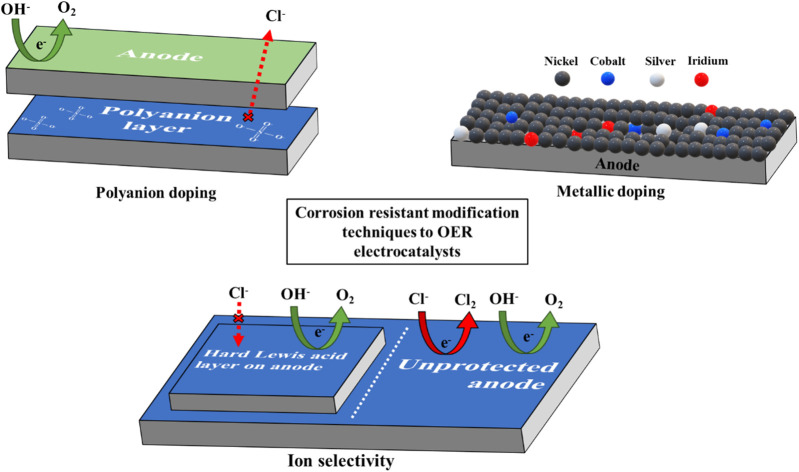

## 1. Introduction

The intermittent nature of renewable energy poses a significant challenge to grid stability. An energy storage system is necessary to bridge the gap between power generation and demand, enhancing energy system resilience and cost efficiency. Hydrogen holds immense potential for decarbonising society, with a remarkably high caloric value of 120–142 MJ kg^−1^, about 2.5 times that of fossil fuels.^1,2^ Conventionally, hydrogen is produced by an extensive process plant that reforms hydrocarbons to hydrogen, and up to 99% of hydrogen produced today comes from this method.^3^ In contrast, water electrolysis provides a clean route to hydrogen from water without the consumption of fossil fuels or the emission of CO_2_. If the electricity comes from renewable energy sources, water electrolysis becomes a truly green technology.^4,5^

Existing water electrolysis plants consist of stacks of multiple cells with an aqueous alkaline electrolyte and a porous separator. The maximum current density for water electrolysis is usually around 0.25 A cm^−2^, and the energy efficiency is only about ∼60%.^4^ To overcome these limitations, solid polymer electrolyte (SPE) water electrolysers have been developed.^4,5^ SPE electrolysers that operate in acidic conditions significantly improve conversion efficiency and are available commercially in small units.^4^ However, the hydrogen produced is still expensive due to the heavy dependence on precious metals as catalysts and the use of costly Nafion® membranes. For hydrogen produced through water electrolysis to be cost-competitive, reducing reliance on precious metals and expensive membranes is essential, making it easier to adopt renewable energy sources.^4^

Renewable energy sources can be constrained by social and spatial factors.^6,7^ Offshore wind applications are favoured for green hydrogen production to minimise electrical infrastructure. However, challenges arise with the expansion of wind farms in scale and number, leading to an increased size and complexity of cable infrastructure.^8^ Converting offshore wind energy to hydrogen at scale is viable, contingent upon growing hydrogen demand in regions housing offshore wind capacity.^8,9^ Gas pipelines entail notably lower investment costs than electricity cables, and preexisting oil and gas infrastructure could be repurposed for hydrogen transport.^8,10^ Around 600 oil and gas facilities and 10 000 km of offshore pipelines in the North Sea could be decommissioned and repurposed for hydrogen transport.^8,11^ However, if conventional electrolysers were to be used, water desalination would be required. A recent analysis by Hausmann *et al.*^12^ argues that on the premise of efficiency, current capital and operational expenditure (CAPEX and OPEX) of incumbent desalination technology (reverse osmosis (RO)), the impact of purifying seawater is negligible and only increases the total cost of the hydrogen produced by 1%.^12^ This appears to be a case-closed type analysis; however, caution is required; the authors mention the complexity of comparing a highly mature technology and a technology in the early stages of development. It is essential to highlight that while desalination accounts for a small percentage of cost, in comparison to the CAPEX of proton exchange membrane (PEM) electrolysers, it is estimated that the CAPEX of electrolysers will reduce by 60–64% by 2025 and 68–72% by 2030,^13^ specifically with the rise of anion exchange membranes (AEMs), that utilise non-precious metals for catalysts, this cost disparity will decrease, and the cost of a desalinated water source will account for a more significant portion of the system, reducing the cost-benefit. Furthermore, the cost of desalinated water is approx. 0.2–3.2 $ per m^3^ using conventional energy sources, but this would appear counterintuitive when trying to create ‘green hydrogen’; thus, using renewable sources for the energy, the price increases to 4–11 $ per m^3^ for desalinated water.^12^ Moreover, the purity of water required for commercial electrolysers is exceptionally high (impurities <10 ppm), which demands repeated processing from RO, which escalates costs.^12,14,15^ Perhaps the most important factor to mention is the effect of scale; RO plants are large installations, producing from 10 000 m^3^ to 1 million m^3^ of water per day.^16^ This is because the CAPEX of RO plants is heavily dependent on plant size due to economies of scale.^16,17^ The largest commercial electrolysers are approximately 10 MW and require 125 m^3^ of water per day, demonstrating a significant mismatch and constructing smaller, dedicated desalination plants for non-centralized hydrogen production is even less viable.^17^

**Fig. 1:**
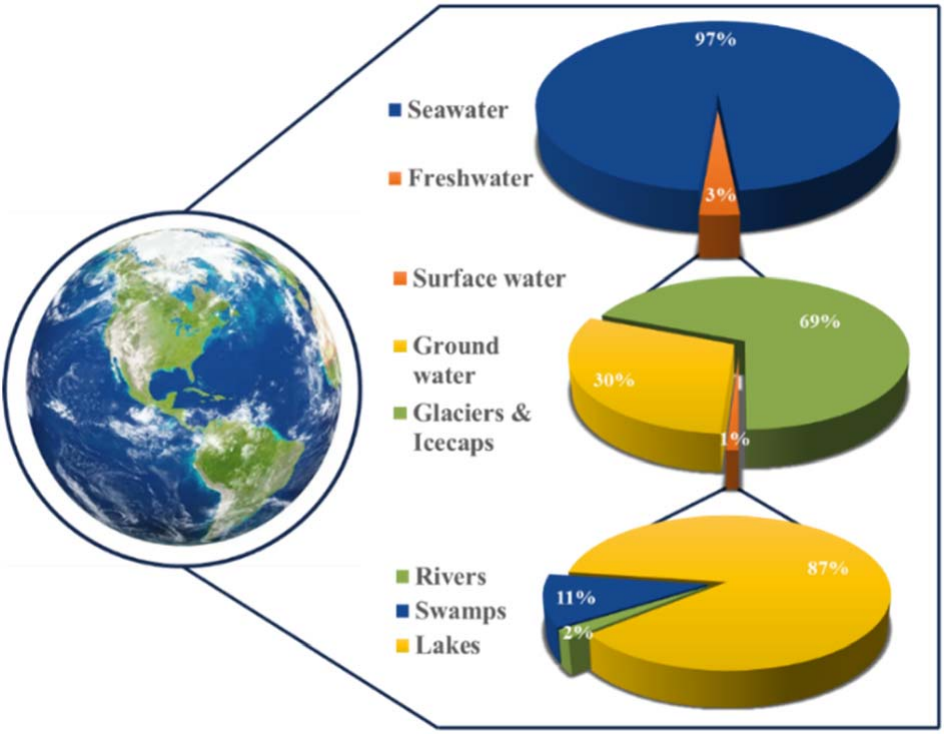
Schematic illustration of global water resources.

The direct electrolysis of seawater offers a range of benefits, from reduced costs due to a simplified system to potential dormant metal recovery,^18^ but corrosion and low hydrogen purity challenges persist.^18,19^ Water electrolysis technologies such as PEM and alkaline water electrolysis (AWE) require ultra-purified water, a concern when scaling production.^20,21^ Freshwater constitutes only 3% of Earth's water, with seawater comprising 97% (Fig. 1). Seawater's complex composition hinders the maturation of direct seawater electrolysis (DSWE) technologies. PEM water electrolysis outperforms DSWE, but introducing water with impurities greater than 300 ppm into PEM systems can lead to noble metal catalyst poisoning and stack failure.^20,22^ As a result, DSWE provides an attractive alternative to freshwater electrolysis by overcoming these challenges, alleviating global freshwater demand and tapping into an almost boundless fuel source.

Seawater's high chloride concentration (0.5 mol dm^−3^) can trigger side reactions such as the chlorine evolution reaction (ClER) that compete with the desired anodic processes, thus impacting electrolyser performance and creating toxic chlorine products.^23^ Additionally, seawater's abundance of cations such as magnesium and calcium can precipitate as hydroxides on the surface of the cathode; prolonged exposure raises the pH at the cathode surface, leading to deposit accumulation that obstructs gas evolution and poisons the electrocatalyst.^24^ Moreover, the presence of bacteria and microorganisms in natural seawater can lead to the poisoning of electrodes and membranes, thereby limiting long-term stability.^14^ To be viable, DSWE must show industrial-scale attributes, including current densities >1 A cm^−2^, cell voltages below 2 V, and stack stability >60 000 hours.^12^

In recent years, there has been a rapid growth in scientific reports concerning seawater electrolysis,^25^ representing an active area of scientific investigation. A substantial increase in corresponding review papers mirrors this trend. Notably, attention from reviewers has predominantly centred on electrocatalysts for the oxygen evolution reaction (OER), driven by the intricate anodic chemistry, with some addressing this topic exclusively.^25,26^ Nevertheless, most review papers encompass a subsequent section dedicated to hydrogen evolution reaction (HER) electrocatalysts, exploring similar chemical attributes concerning hydrogen generation in seawater.^1,14,27–36^ These review papers exhibit a common structural framework, often featuring four main discussion sections. Of the four main sections, analysis of types of compounds (*e.g.* sulphides, phosphides and oxides/hydroxides) and unique design criteria of electrocatalysts (hierarchical structure and corrosion resistant layers) are the most popular structures and what we summarise predominantly in this review. The most popular structure reviewers have focused on discussing is the general design criterion of electrocatalysts for both OER and HER, with chapters exploring the pH design criterion, Cl^−^ blocking layers, local reaction environment, surface wettability and selective OER sites of electrocatalysts.^1,14,26,28,30,32,34–39^ Reviews on types of compound combinations such as metal phosphides/phosphates, metal nitrides, metal dichalcogenides, metal oxides, hydroxides and (oxy)hydroxides are explored widely.^1,19,27–29,33,40,41^ Subsequent review papers^1,28,29,31,33,41,42^ split electrocatalysts into abundancy, with a focus on earth-abundant electrocatalysts for seawater splitting, moving away from precious metal and platinum group metals, which are limited in applications by cost. A few reviews focused on electrolyte conditions instead of catalyst development, analysing the impact of varying pH.^25,43^

This review investigates state-of-the-art OER electrocatalysts for seawater electrolysis capable of operating at current densities ≥100 mA cm^−2^ for hydrogen production. The review aims to add novelty by investigating modification techniques not seen in the majority of review papers (ion selectivity) and by adding essential critical evaluation to discuss high-performance of modified OER electrocatalysts for seawater electrolysis, highlighting key parameters of recent research that can lead to the development of low-cost, highly efficient earth-abundant electrocatalysts for direct seawater electrolysis.

## 2. Mechanism of direct seawater electrolysis

### 2.1. General electrochemistry of water electrolysis

The water-splitting reaction requires an external stimulus, *i.e.* a potential difference between two electrodes, to drive the overall cell reaction. Since the average pH for seawater is 8.2, the electrode reactions for water electrolysis in alkaline solutions are most relevant. They are shown in eqn (1) and (2), with the overall reaction in eqn (3). An alkaline electrolyte solution also allows the use of non-precious metal electrocatalysts, which is the main focus of this review.

Cathode, HER

1 4H_2_O_(l)_ + 4e^−^ → 2H_2(g)_ + 4OH^−^_(aq)_, *E*^0^ = −0.83 V *vs.* standard hydrogen electrode (SHE)

Anode, OER

2 4OH^−^_(aq)_ → O_2(g)_ + 2H_2_O_(l)_ + 4e^−^, *E*^0^ = +0.40 V *vs.* SHE

Overall cell reaction

3 2H_2_O_(l)_ → 2H_2(g)_ + O_2(g)_, *E*^0^ = 1.23 V

Under standard conditions, a minimum potential of 1.23 V is required to commence the decomposition of water into H_2_ and O_2_.^44^ The standard enthalpy, Δ*H*^o^, for reaction (3) is +286 kJ mol^−1^ of H_2_ and the Gibbs energy, Δ*G*^o^, is +238 kJ mol^−1^ of H_2_.^44^

In practice, the cell voltage to drive the water electrolysis reaction is given by eqn (4).

4 −*E*_cell_ = Δ*E*_e_ − |*η*_a_| − |*η*_c_|−*IR*

where Δ*E*_e_ is the difference in the equilibrium potentials for the two electrode reactions (1.23 V), and the other terms are inefficiencies that lead to increased energy consumption and should, therefore, be minimised; *η*_a_ and *η*_c_ are the overpotentials at the anode and cathode, respectively while *IR* is the ohmic losses due to current (*I*) flowing through the cell with resistance *R*. The *η* terms can be minimised using high-performance electrocatalysts, while minimising the *IR* term depends on good electrochemical engineering.^44^

The reaction kinetics of both anodic and cathodic reactions depend greatly on the electrocatalyst used. The anodic overpotential for OER is significantly larger than the cathodic overpotential and is a significant source of energy loss in water electrolysis cells. Hence, reducing anodic overpotential is the critical target in alkaline water electrolysis. This review will discuss OER electrocatalysts in conditions appropriate to DSWE.

### 2.2. Competition between OER and ClER

**Fig. 2:**
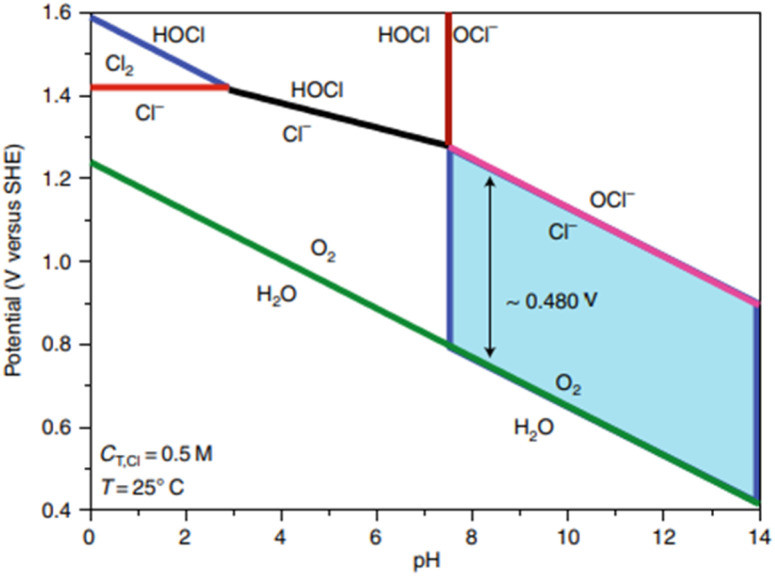
Pourbaix diagram demonstrating the trade-off between OER and chloride chemistry. Adapted from ref. 24 with permission from American Chemical Society, copyright 2019.

Seawater composition constitutes primarily water with 3.5% salts by weight. Of the dissolved ions, chloride (Cl^−^) accounts for 55.04%, followed by sodium (Na^+^) at 30.61%; this is why approximately 0.5 M NaCl solution is commonly employed in simulated seawater.^34^ Other ions such as sulfate (7.76%), magnesium (3.69%), calcium (1.16%), potassium (1.10%), bicarbonate (0.41%), bromide (0.19%), borate (0.07%) and strontium (0.04%) can interfere with reactions at either electrode. However, the effect of Cl^−^ is the primary focus here due to its high concentration, causing competing reactions to the desired evolution of oxygen. The competition between chlorine evolution and oxygen evolution can be represented as a Pourbaix diagram (Fig. 2).

At pH 0,

5 2Cl^−^ → Cl_2_ + 2e^−^, *E*^0^ = 1.36 V *vs.* reversible hydrogen electrode (RHE), ClER

At pH 14,

6 Cl^−^ + 2OH^−^ → ClO^−^ + H_2_O + 2e^−^, *E*^0^ = 1.72 V *vs.* RHE, ClOR

Chloride electro-oxidation chemistry is complex, with different reactions occurring depending on the pH, temperature, and applied potential. Fig. 2 demonstrates the potential-pH zones where the OER and chloride oxidation reactions become thermodynamically possible. For simplicity, a temperature of 298 K is considered a standard temperature, with a chloride concentration at 0.5 M (*C*_T, Cl_), a typical chloride salt concentration in seawater. The green line illustrates the thermodynamic equilibrium before water decomposes to oxygen. The OER reaction is thermodynamically favourable if electrode potentials are more positive than the green line, and chlorine oxidation is favoured if the potential is above the pink line (eqn (6)). Therefore, there is a potential window of ∼480 mV for an alkaline environment where oxygen evolution is possible without the oxidation of chlorine, as shown in the blue highlighted area. The red line demonstrates the competition between the chlorine oxidation reaction (ClOR) and the gaseous evolution of chlorine (eqn (5)).^45^

## 3. Challenges to seawater splitting

### 3.1. General challenges

**Fig. 3:**
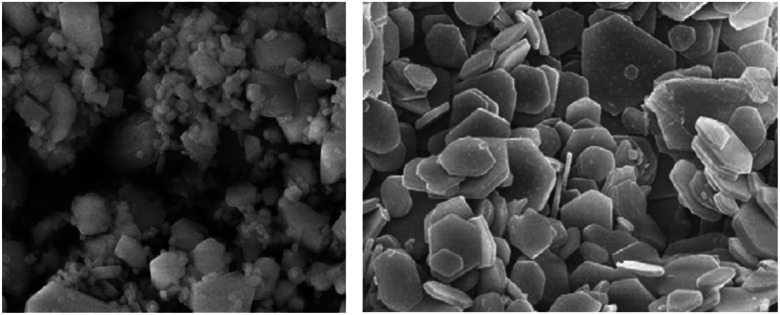
Scanning electron microscope image of Mg(OH)_2_ salt precipitation deposits, adapted from ref. 47 with permission from Elsevier, copyright 2009.

Electrolysis in seawater can be complicated at the cathode due to the interference of ions and pH fluctuations, which hinders reactions.^24^ Unbuffered seawater has slower kinetics for the HER, which can lead to local pH changes at the cathode surface, causing the precipitation of dissolved ions.^33,38^ When the pH fluctuates above 9.5, it can result in catalyst degradation and the precipitation of magnesium hydroxide (Fig. 3) and calcium hydroxide, obstructing active sites and reducing the electrode activity. Salt deposits, microbes, bacteria, and small particles can also be challenging to eliminate but can be minimised by introducing turbulence, supporting electrolytes, and selecting appropriate catalysts and current densities.^14,24,46^

While extensive research has been conducted on seawater salts, limited data is available on microorganisms and their implications on DSWE.^25^ Existing studies on microorganisms lack comprehensive investigations, making this aspect relatively underexplored. Studies comparing water splitting in simulated seawater to actual seawater note a decrease in the current density achieved or an increase in overpotential but fail to explain the precise chemistry causing the reduced performance. In seawater electrolysis, biofouling is a primary challenge that can lead to active site blockage, membrane complications, and reduced equipment lifespan.^25,48^ Despite efforts to mitigate this issue, implementing direct seawater applications may introduce complications, such as the necessity for multiple coatings on electrodes, potentially affecting catalytic performance.^25,49,50^

### 3.2. Challenges in terms of OER catalysts

**Fig. 4:**
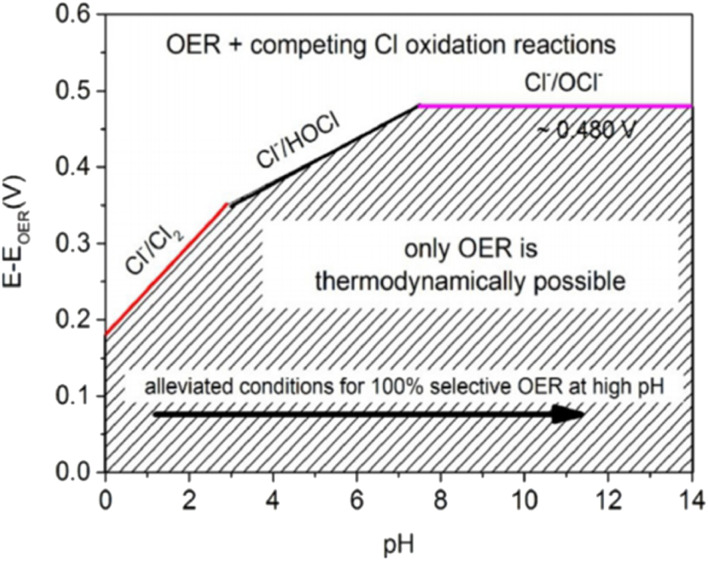
Maximum permitted overpotentials for OER electrocatalysts, reproduced from ref. 54 with permission from John Wiley and Sons, copyright 2016.^54^

To efficiently produce H_2_ through DSWE, a highly selective OER catalyst is required. Increasing the pH of seawater by adding potassium hydroxide (KOH) has been shown to aid reaction selectivity at the anode and increase the potential region for OER (Fig. 4).^51–54^

Under highly alkaline conditions, the product of the ClER is altered from hypochlorous acid to hypochlorite, denoted as the chlorine oxidation reaction (ClOR) (eqn (7)). ClO^−^ also has a kinetic advantage over the OER under standard conditions (25 °C and 0.5 M), but the OER is still more thermodynamically favourable.^54,55^ The standard hypochlorite redox potential is heavily influenced by pH; the gradient of the slope of pH dependency is identical to the OER potential slope in the Pourbaix diagram (Fig. 2).^55^ The potential for ClO^−^ formation is ∼480 mV higher than the potential for OER in alkaline solutions.^52^ Therefore, if electrocatalytic oxidation can operate at less than 1.72 V_RHE_ for complete seawater electrolysis and at an overpotential of the OER less than 480 mV in alkaline electrolytes, theoretically, no hypochlorite is formed, as it is thermodynamically suppressed in this potential region and approximately 100% selectivity will be achieved.^52,56^ This is how the activity of OER electrocatalysts is measured in DSWE literature, the overpotential in relation to the theoretical threshold of 480 mV.

As a result, a design criterion is proposed for selective OER control, where at pH greater than 7.5, the reaction is given:^55^

7 *η*_OER_ < 480 mV, at pH > 7.5

Fig. 4 shows that for a 100% selective OER region below 480 mV, pH must be at least 7.5; decreasing pH reduces the potential required for ClOR to compete with OER. Anode catalysts must be highly selective to minimise the creation of highly corrosive hypochlorite during seawater electrolysis.^14^

## 4. Different OER catalysts

### 4.1. Strategy to design OER catalysts for DSWE

The pH of natural seawater depends on depth, latitude, and other conditions. However, it is widely considered to be in the range of pH 8 to 8.3,^57^ classifying it as an alkaline solution. The design criteria for alkaline OER catalysts are based on thermodynamic and kinetic considerations, and saline water is a non-buffered electrolyte. Applying an additive (1 mol dm ^−3^ of KOH) is commonly used to prevent changes in the local pH during electrolysis and aid ion selectivity. Nickel is widely recognised for its excellent OER activity and significant corrosion–resistant properties in alkaline solutions, making it an ideal component of electrode materials for use at various levels of alkalinity and temperature conditions.^58^ Nickel and its alloys possess a desirable suite of characteristics, rendering them highly suitable for deployment in seawater (alkaline) environments, whether as the primary catalyst material or as the substrate. Nickel is a cost-effective electrode material relative to platinum group metals (PGMs) and possesses good electrical conductivity due to its loosely bound valence electrons.

### 4.2. Nickel and iron layered double hydroxides (LDHs)

**Fig. 5:**
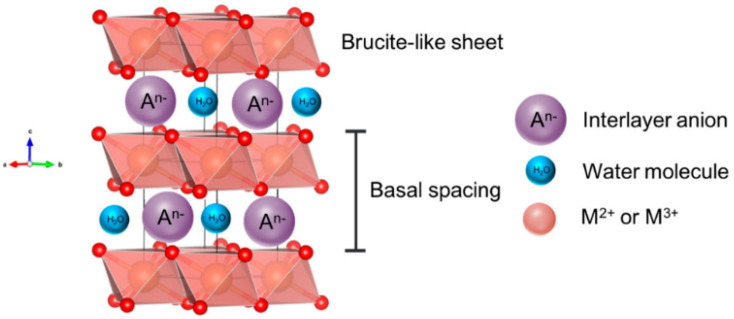
Schematic showing the structure of a layered double hydroxide (LDH), reproduced from ref. 59 under Creative Commons License (CC BY).

Nickel LDHs are becoming ever more popular electrocatalysts for alkaline seawater electrolysis. LDHs are materials that exhibit an ultrathin two-dimensional structure of brucite-like layer stacking^59^ (Fig. 5) and are characterised by high porosity and the presence of versatile anionic particles that can be easily modified and exchanged within the basal spaces.^60–62^ Positively charged divalent cations such as Ni^2+^, Mn^2+^, Co^2+^, Cu^2+^, and Mg^2+^ construct positively charged layers while intercalated anions can be easily modified to SO_4_^2−^, Br^−^ or PO_4_^3−^.^63^ A key attribute of LDHs lies in their ability to retain the interlayer spaces, allowing for the effective accommodation of a diverse range of anionic species.^60^ This inherent flexibility makes LDHs well-suited for various applications, including DSWE, specifically for the electrostatic repulsion strategy to mitigate chloride corrosion.^60^

**Fig. 6:**
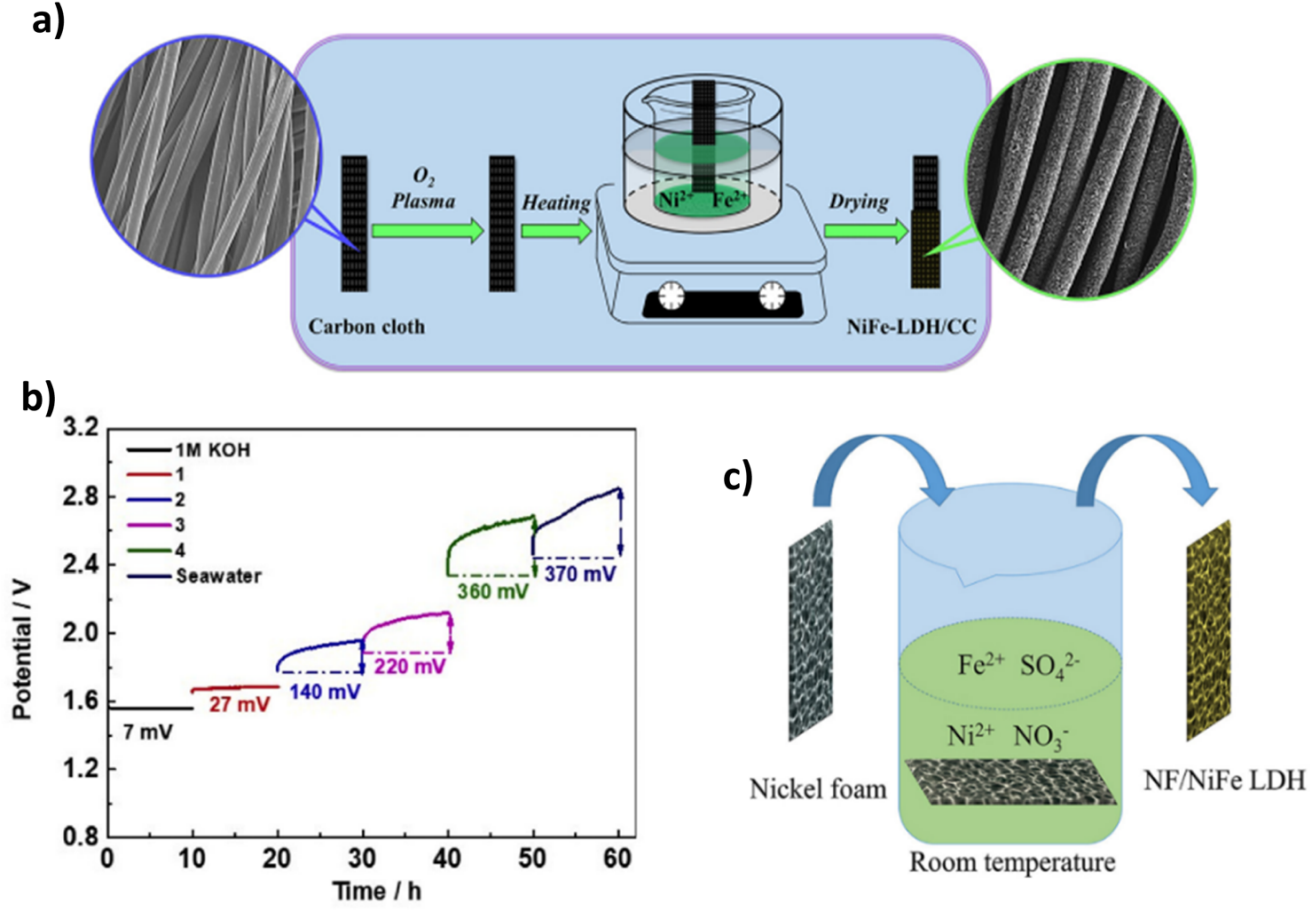
(a) NiFe-LDH-6-4/CC synthesis procedure, reproduced from ref. 64 with permission from Elsevier, copyright 2021. (b) Chronopotentiometry test of NiFe LDH/CC in 1 M KOH, 1 (7.5 ml KOH & 7.5 ml seawater), 2 (3.4 ml KOH & 11.6 ml seawater), 3 (1.7 ml KOH & 13.3 ml seawater), 4 (0.9 ml KOH & 14.1 ml seawater) & seawater, reproduced from ref. 65 with permission from Elsevier, copyright 2021. (c) Mechanism for spontaneous growth of NiFe LDH at room temperature on NF, reproduced from ref. 66 with permission from Elsevier, copyright 2021.

Table 1 lists some of the best performing LDH DSWE catalysts. Using carbon cloth (CC) as a substrate is common throughout the literature due to the excellent catalyst substrate contact created by abundant nano-to-microscale pores on the CC surface, offering significant area for electrochemical reactions and electrolyte interactions.^64^ However, carbon is unstable at high anodic potentials because the standard potential for carbon oxidation is 0.207 V *vs.* RHE, meaning carbon corrosion is expected at elevated anodic potentials and inevitably leads to electrode deterioration.^67^ Dong *et al.* reported a range of NiFe-LDHs on carbon fibre cloth with varying atomic ratios. The NiFe-LDH-6-4/CC was fabricated simply on a CC by application of mild chemical methods conducted under atmospheric pressure and temperature lower than 100 °C (Fig. 6a).^64^ Since NiFe-LDHs are a well-established OER catalyst, the ratio of nickel to iron has been a research focus; a lot of studies are in agreement with Li *et al.*^68^ who identified that small additions of iron in the composition of nickel to iron, enhanced the activity and rate of OER. In contrast, more significant additions counteracted the OER activity, and where iron additions outweigh nickel, the performance is worse than pure nickel; Dong *et al.* further confirm this point.^64,68^ A nickel-to-Fe ratio of 6 : 4 demonstrated the best activity, performance and stability in 1 M KOH & seawater, affording an overpotential of 301 mV at 100 mA cm^−2^ and remaining consistently at that potential for 165 h.^64^ By increasing the content of nickel, higher activity and a smaller overpotential are achieved owing to nickel's high conductivity and the redistribution of nickel and Fe atoms in the catalyst, which bonds O^2−^ and increases the electrochemical active sites.^64,69,70^

**Table 1:** OER electrocatalysts with different substrates and corresponding performance in saline electrolytes

OER catalyst	Ref.	Duration (h)	Electrolyte	Cell voltage (V)	Current density (mA cm^−2^)	Overpotentials to achieve current density: *η* (mV)
NiFe-LDH/CC	64	165	1 M KOH + seawater	1.57	100	301
NiFe-LDH/CC	65	10	1 M KOH + seawater	2	100	1 = 27
						2 = 140
						3 = 220
						4 = 360
NiFe-LDH/NF	66	100	1 M KOH + seawater	1.533, 1.665	100 & 500	247, 296

Lu *et al.* also investigated a NiFe-LDH on CC, denoted (NiFe-LDH/CC). It is synthesised using a two-step hydrothermal method to grow “sheet-shaped” NiFe hydroxide on the CC support.^65^ NiFe-LDH/CC is tested systematically in various electrolyte conditions with varying amounts of KOH and seawater in each stability test. Ten-hours constant current tests were conducted during this study at a current density of 100 mA cm^−2^. The results indicate that as the concentration of KOH decreases and the volume of seawater increases, a greater overpotential is presented,^65^ as would be anticipated (Fig. 6b). At 100 mA cm^−2^, in pure seawater (no additional buffer), NiFe-LDH/CC exhibited an overpotential rise of 370 mV over the period, indicating a degradation rate of 37 mV h^−1^, significantly higher than comparable catalysts,^71,72^ showing further overpotentials with an extended test, would shift this catalyst into the region of competition between ClOR and OER. There is no mention from the authors what specific component caused this, but it is likely there was corrosion of the substrate due to carbon's instability at higher anodic potentials. However, this is operating in pure seawater. Thus, the conductivity of the solution will be significantly less. Overall, it is beneficial to the research area to learn about the influence of varying concentrations of seawater and the implication on one of the better-performing earth-abundant OER electrocatalysts (NiFe-LDH).

Nickel foam (NF) is a commonly used substrate material in literature due to its plentiful active sites and 3D hierarchical structure with high porosity and a suitable catalyst substrate connection, even simply dipping within a solution.^66^ Ning *et al.* reported a NiFe-LDH on an NF substrate (Fig. 6c). NiFe-LDH is synthesised *via* immersion in a solution at room temperature for a period ranging from 1 to 5 hours to create NiFe-LDH on the substrate.^66^ The one-step spontaneous reaction for NiFe-LDH deposition is facile and time-effective, but it could be argued that there will be a fragile bond between the catalyst and substrate *via* this synthesis. The NiFe-LDH nanosheets are a product of the oxidation of Fe^2+^ ions, an aspect that is typically avoided in electrodeposition techniques. However, this study utilises the Fe^2+^ to Fe^3+^ oxidation to create an exceedingly active OER electrocatalyst.^66,73,74^ The tested electrolyte compositions include 1 M KOH, 1 M KOH, 0.5 M NaCl, 1 M KOH, 1 M NaCl, and 1 M KOH and seawater. Similar to most NiFe-LDH catalysts reported in the literature, the catalyst's performance is good, with current densities of 100 mA cm^−2^ and 500 mA cm^−2^ achieved with overpotentials of 247 mV and 296 mV, respectively. It was noted that activity within the seawater electrolyte was lower due to the poisoning effect of the impurities within seawater,^66^ an aspect not considered when using simulated seawater as an electrolyte. Stability is also of utmost importance in catalyst development, NiFe-LDH/NF reveals good performance over a 100 h test in varying electrolytes, even at current densities of 500 mA cm^−2^, demonstrating its potential for industry applications.^66^

### 4.3. Nickel and iron mixed metal oxides

**Fig. 7:**
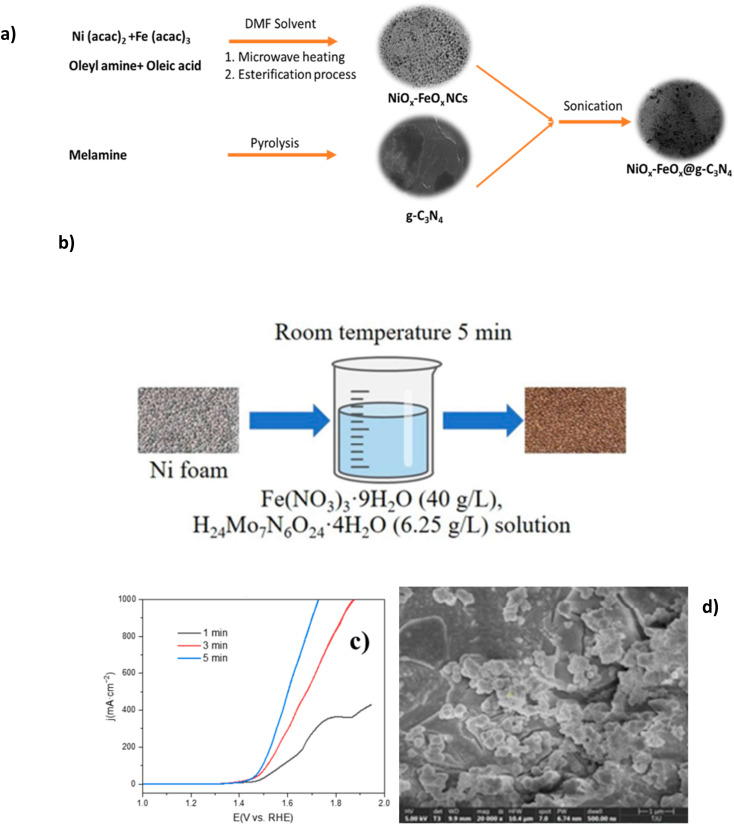
(a) Systematic synthesis of NiO_*x*_-FeO_*x*_@g-C_3_N_4_, reproduced from ref. 77 with permission from American Chemical Society, copyright 2022. (b) Synthesis procedure of (Ni/Fe/Mo)OOH, (c) polarization curves of electrochemical performance of varied synthesis immersion times of (Ni/Fe/Mo)OOH, (d) SEM images of (Ni/Fe/Mo)OOH after 5 minutes of immersion synthesis. (b)–(d) Are reproduced from ref. 79 under Creative Commons License (CC BY).

Mixed metal oxides have recently proven excellent performance in seawater electrolytes. Studies have investigated the creation of various metal oxide compounds to exploit the synergetic effect of different metal species and optimise corrosion prevention while improving OER performance.^56,75,76^ Ul Haq *et al.* synthesised a novel structure of graphitic carbon nitride-supported nickel-iron oxide (NiO_*x*_-FeO_*x*_@g-C_3_N_4_). Synthesis of NiO_*x*_-FeO_*x*_ (Fig. 7a) is prepared using Ni^2+^ bis(acetylacetonate) and Fe^3+^ tris(acetylacetonate), often abbreviated as Ni_3_(acac)_2_ and Fe(acac)_3_, respectively, in the presence of oleyl amine and oleic acid.^77^ The oleylamine controlled the nucleation rate and acted as a reducing agent, while the oleic acid was responsible for bonding metal ions to the substrate, creating homogeneous growth of nanoclusters (NCs) and acting as the capping agent, as a result leading to a smoother catalyst morphology. The N-doped carbon was selected due to its desirable corrosion resistance and tunable surface chemistry.^77,78^ The synthesis of the NiO_*x*_-FeO_*x*_@g-C_3_N_4_ is complex, consisting of five different in-depth processes, which may be a barrier to scaling up. 380 mV overpotential was required to achieve a current density of 1000 mA cm^−2^, with sustained performance for more than 100 hours in 1 M KOH + seawater at ambient temperature while the formation of hypochlorite was suppressed.^77^ This corrosion resistance can be linked to the N-doped carbon (g-C_3_N_4_) support, which protects from stress and pitting corrosion by forming π- and δ-bonds between the nuclei of carbon and N atoms while reducing interfacial resistance amongst OER intermediates and active sites.^77^ Experimental evidence revealed no substrate oxidation occurred with the g-C_3_N_4_ support while preserving the active sites.^77^

NiO_*x*_-FeO_*x*_@g-C_3_N_4_ provides valuable insights for the future development of OER electrocatalysts and could serve as valuable research for further investigation, particularly because of the performance achieved in natural seawater electrolyte. This study's graphitic carbon nitride support provides valuable analysis for further work to build upon and demonstrates significant corrosion benefits within a seawater environment.

Table 2 shows some high performance mixed metal oxide DSWE catalysts. This further confirms the benefit of mixed metal oxides for alkaline seawater splitting and emphasises the importance of simplicity when synthesising catalysts. Xu *et al.*^79^ in 2023 synthesised a (Ni/Fe/Mo) (oxy)hydroxides (OOH) catalyst on a nickel foam (NF) substrate *via* a simple, low-cost, one-step immersion synthesis at room temperature. The one-step synthesis (Fig. 7b) requires only a 5 minutes immersion in easily obtainable and cost-effective reagents (Fig. 7b); this study aims to prove that complex and expensive equipment is not necessary for creating highly active and stable electrocatalysts for seawater electrolysis. NF is used as the source of nickel and as the substrate to grow the (Ni/Fe/Mo)OOH. Despite the limited immersion time, catalyst coverage on the substrate is good, with cluster structure diameter ranging from 1 μm to 10 μm and the morphology is relatively uniform (Fig. 7d). The authors observe a trend that increasing the immersion time (Fig. 7c), results in greater catalyst attachment on the substrate framework, however, it is not clear why a longer immersion than 5 minutes wasn't used to increase the amount of catalyst on the substrate. OER performance of (Ni/Fe/Mo)OOH was investigated in 1 M KOH and seawater, where it presented overpotentials of 330, 416 and 514 mV at 100, 400 and 1000 mA cm^−2^, respectively. Comparably higher overpotentials than NiO_*x*_-FeO_*x*_@g-C_3_N_4_ and a concerningly high overpotential at 1000 mA cm^−2^ of 514 mV, operating in a region where the ClOR can evolve readily. Further to this, the catalyst can operate stably in 1 M KOH and seawater at 100 mA cm^−2^ for 72 h, with only an increase in voltage of 10 mV, indicating a 0.14 mV h^−1^ degradation rate, which is relatively low compared to other electrocatalysts analysed in this review and impressive considering the simplicity and efficiency of synthesis. This work is an insightful and valuable study for further research on simple, cheap and effective OER electrocatalysts. A lot of benefits can be derived from such a simple synthesis; considering the impressive performance of an alkaline seawater electrolyte, more effort is needed to improve the conductivity and activity of the catalyst to reduce the overpotential at higher current densities.

**Table 2:** Mixed metal oxides as OER electrocatalysts for DSWE

OER catalyst	Ref.	Duration (h)	Electrolyte solution	Current density (mA cm^**−**2^)	Overpotentials to achieve current density: *η* (mV)
NiO_*x*_-FeO_*x*_@g-C_3_N_4_	77	>100	1 M KOH + seawater	1000	380
(Ni/Fe/Mo)OOH	79	72	1 M KOH + seawater	1000	514

### 4.4. Modification techniques

#### 4.4.1. Doping

Doping, whether metallic or anionic, is an increasingly popular technique for improving catalytic performance, stability and corrosion protection. Metallic doping is typically used to enhance the catalytic properties of electrocatalysts, specifically doping precious metals with earth-abundant elements to enhance conductivity.^56,72,80–82^ Anionic doping is commonly used to improve the stability and corrosion resistance to Cl^−^ ions within seawater due to the negative charge that repels Cl^−^.

##### 4.4.1.1. Metallic dopants (Co, Mn, Ag, Ir)

State-of-the-art electrocatalysts commonly comprise platinum group metals like iridium, platinum, and palladium. These are valued for their low overpotentials and Tafel slopes, particularly in acidic conditions.^63,83,84^ However, the widespread commercial use of these metals is limited due to their high cost and scarcity.^85,86^ Subsequently, research has focused on reducing the precious metal loadings in electrocatalysts for seawater splitting, aiming to achieve cost-effective solutions. Combining platinum group metals with nickel can help tune electronic structures and improve charge transfer, exposing more active sites and reducing costs. Table 3 lists some examples of metallic doped OER catalysts.

**Table 3:** Metallic doped OER electrocatalysts and corresponding performance in saline electrolytes

Dopant	OER catalyst	Ref.	Duration (h)	Electrolyte solution	Current density (mA cm^−2^)	Overpotentials to achieve current density: *η* (mV)
Silver (Ag)	Ag/NiFe LDH	80	1000	1 M KOH + seawater	1000	303
Iridium (Ir)	NiIr-LDH	81	650	1 M KOH + seawater	500	361
Cobalt (Co)	NiFe-CuCo LDH	72	500	6 M KOH + seawater	500	283
Manganese (Mn)	Mn-Ni_2_P-Fe_2_P	82	200	1 M KOH + 0.5 M NaCl	500 + 1000	325@500
						358@1000

Precious metal doping of transition metal LDHs is an increasingly common research area for creating efficient OER electrocatalysts and reducing precious metal loading. Ag doping has been found to increase abundant active sites and improve electron transfer, enhancing OER activity.^80^ Liu *et al.* synthesised a NiFe-LDH catalyst supported by Ag *via* a one-step redox reaction on nickel foam, where Ag was supported on top of a NiFe-LDH catalyst. Ag incorporation increased the phase stability of the NiFe-LDH, and any exposed Ag nanowire operated as active sites, helping to release OH^−^ adsorbates from the active sites. Using Ag as a dopant enables the catalyst to reach the current densities necessary for industrial applications, which is why the authors selected Ag. The Ag/NiFe-LDH demonstrated improved conductivity, increased number of active sites and enhanced surface area compared to NiFe-LDH.^80^ Ag/NiFe-LDH showed excellent durability with an operation of 1000 hours in alkaline natural seawater (1 M KOH + seawater) with a small overpotential of 303 mV at 1000 mA cm^−2^ and can serve as a valuable touchstone for future work.

Introducing a 5d precious metal, iridium, to a Ni-LDH achieves better electron transfer performance as the electron interaction between nickel and Ir optimises electron structure.^81,87^ You *et al.* introduced iridium to Ni-LDH to form a NiIr-LDH monolayer.^81^ NiIr-LDH was synthesised through a coprecipitation process using metal precursors in formamide. NiIr-LDH showed improved performance in both alkaline simulated and natural seawater with overpotentials of 286 mV and 315 mV, respectively, to reach 100 mA cm^−2^ and 361 mV to reach 500 mA cm^−2^ in alkaline natural seawater. In contrast, a commercial IrO_2_ catalyst required 763 mV overpotential at 500 mA cm^−2^ under the same conditions.^81^ Stability was also significantly enhanced by introducing Ir, as the catalyst remained stable at 500 mA cm^−2^ for 650 hours. Adding precious metals to nickel-based catalysts has increased OER activity, with both Liu *et al.* and You *et al.* reporting catalysts that can reach 500 mA cm^−2^ with low overpotentials.

**Fig. 8:**
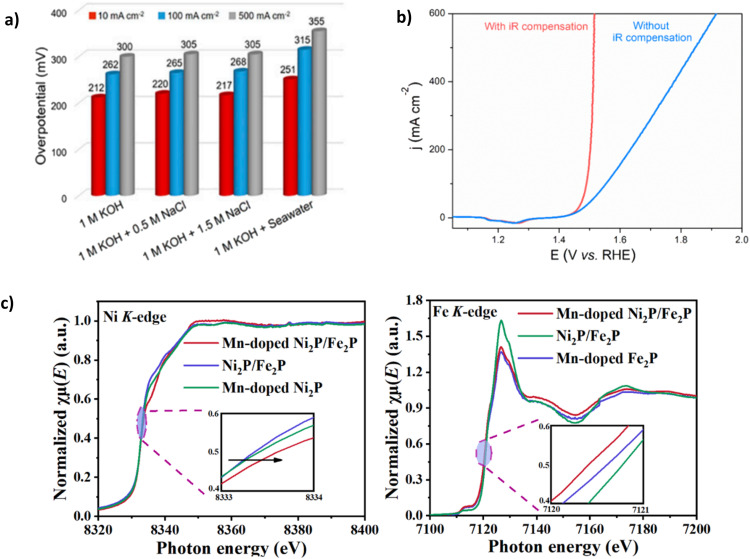
(a) NiFe-CuCo-LDH performance in varying electrolytes (b) *IR* compensation applied to OER polarisation curves of NiFe-CuCo-LDH, reproduced from ref. 72 under Creative Commons Attribution-NonCommercial-NoDerivatives License 4.0 (CC BY-NC-ND). (c) Ni and Fe K-edge X-ray absorption near-edge spectroscopy (XANES) spectra of Mn-Ni_2_P-Fe_2_P, reproduced from ref. 82 with permission from Elsevier, copyright 2023.

Despite its natural abundance, cobalt has been included in this category due to higher supply risk in metal criticality studies, extraction complexities and projected future demand, ultimately leading to increased costs.^88,89^ Nickel-based catalysts have been increasingly combined with cobalt due to enhanced surface redox attributes.^90^ Yu *et al.* demonstrated the benefit of using Co by synthesising NiFe-CuCo LDH.^72^ Using a facile and time-effective approach. NiFe-CuCo LDH illustrated good stability at 500 mA cm^−2^ with an overpotential of 283 mV for 500 hours in 6 M KOH and natural seawater (pH 8.2). Notably, 6 M KOH is a highly concentrated solution, and it is well reported in the literature^76^ that high concentrations of KOH can result in reduced overpotentials.^65^ Thus, it could be argued that the overpotential figures reported are somewhat deflated, particularly as a relatively high *IR* compensation of 85% was used (Fig. 8b). To illustrate this point, for the catalyst to reach 500 mA cm^−2^ in 1 M KOH + seawater (Fig. 8a), an overpotential of 355 mV is required, 72 mV higher than in 6 M KOH + seawater. With only an increase of 18 mV in overpotential over 100 h, a degradation rate of 0.18 mV h^−1^ and 71 mV after a period of 500 h with a degradation rate of 0.142 mV h^−1^, the catalyst undoubtedly exhibits excellent stability, with a decrease in degradation rate over a longer duration. The performance and stability of NiFe-CuCo-LDH can be attributed to the hierarchical structure of the catalyst, abundant exposed active sites stemming from the CuCo-LDH and enhanced charge transfer characteristics and corrosion resistance. Introducing nickel and Fe aided modulation of the electronic structure of CuCo-LDH, helping to improve electrical conductivity and, as a result, charge transfer.^72^ Despite its natural abundance, cobalt has a higher supply risk in metal criticality studies, extraction complexities and projected future demand, ultimately leading to increased costs.^88,89^

Mn doping can generate more active sites and optimise electrocatalysts' electronic structure because of the many different valence states that Mn can exhibit.^82^ Luo *et al.* synthesised an Mn-doped NiFe phosphide, denoted Mn-Ni_2_P-Fe_2_P. Mn doping and NiFe phosphide aid modulation of the electronic structure (Fig. 8c), which is confirmed using X-ray absorption near-edge spectroscopy (XANES), where the K-edge positions of nickel and Fe in the Mn-Ni_2_P-Fe_2_P catalyst shift positively and negatively, respectively upon introduction of Mn.^82^ NiFeMn-layered triple hydroxide (LTH) 3D nanoflowers consisting of self-accumulated 2D nanosheets grown on the substrate *via* the hydrothermal method. A phosphorylation process evolves the structure into 3D nanoflowers and the Mn-Ni_2_P-Fe_2_P catalyst, creating plentiful active sites.^82^ The catalyst can achieve current densities of 500 mA cm^−2^ and 1000 mA cm^−2^ with overpotentials of 325 mV and 358 mV, respectively,^82^ well below the 480 mV threshold for hypochlorite oxidation. However, all electrochemical experiments were conducted under an Ar atmosphere, a widely reported approach in electrocatalyst synthesis but not electrochemical testing. Stability investigations using chronopotentiometry were carried out at 100 mA cm^−2^ and 500 mA cm^−2^ for 200 hours and showed a negligible increase in overpotential over the period, significantly more stable than similarly reported phosphides.^91^

Metallic doping focuses on improving the catalytic activity of the catalyst and therefore improving the performance of the catalyst. A thorough review has revealed that, among other metals, Ag doping has the most significant impact on the performance of a NiFe-LDH catalyst. This catalyst can achieve a remarkable 1 A cm^−2^ at a mere 303 mV and last over 1000 hours in natural alkaline seawater. However, the cost of Ag, currently around £580/kg, poses a challenge in determining the ideal development direction and would pose cost challenges scaling up. Mn doping, on the other hand, offers a better balance between cost and catalytic activity, costing only around £2/kg and achieving 1 A cm^−2^ in simulated seawater. Thus, it presents a better development direction for metallic doping.

##### 4.4.1.2. Electrostatic repulsion

In DSWE studies, it is increasingly common to explore incorporating an embedded repulsion layer that electrostatically repels Cl^−^ ions without affecting the exposed active sites, denoted as the electrostatic repulsion strategy. This section will investigate anionic (S^2−^ and P^3−^) and polyanionic dopants (SO_4_^2−^ and PO_4_^3−^) that are doped into existing highly active OER catalysts.^92^ Extensive analysis in^23,82,91,93–96^ has shown that sulphide doping and phosphide doping (anionic dopants) are very promising approaches to enabling stable performance in the presence of Cl^−^.

###### 4.4.1.2.1. Sulphide doping

**Fig. 9:**
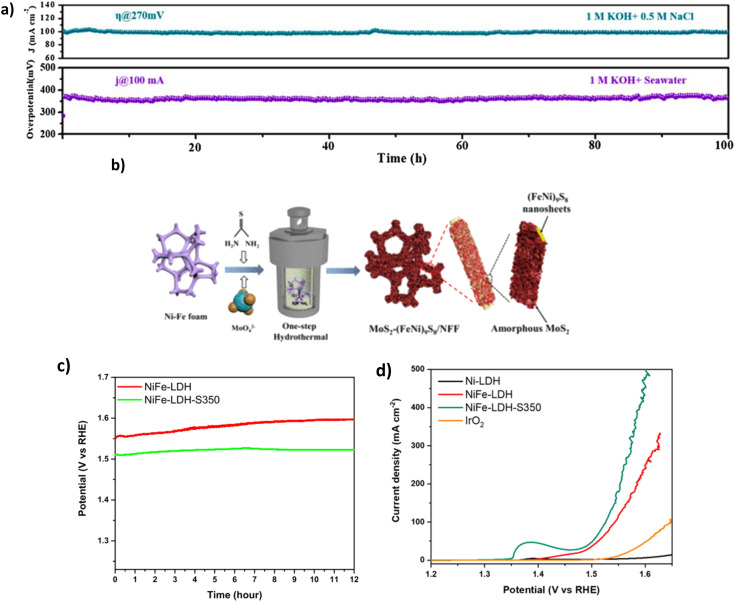
(a) NiCoS stability tests of CP and CA in varying electrolytes, reproduced from ref. 98 with permission from Elsevier, copyright 2021. (b) Synthesis steps of MoS_2_-(FeNi)_9_S_8_/NFF, reproduced from ref. 101 with permission from American Chemical Society, copyright 2022. (c) CP curves at 100 mA cm^−2^ of NiFe-LDH and NiFe-LDH-S-350 in 1 M KOH & 0.5 M NaCl. (d) LSV curve of NiFe-LDH-S-350 in 1 M KOH & 0.5 M NaCl, (c) and (d) are reproduced from ref. 95 with permission from Elsevier, copyright 2021.

Sulphide doping has widely been a disregarded method for OER electrocatalysts for water splitting due to the negatively charged sulphide ion (S^2−^) in its structure, which deters the adsorption of OH^−^ ions to the positively charged anode surface.^97^ Despite this, studies for DSWE have explored the benefit of incorporating an anionic layer as an electrostatic repulsion layer underneath the initial exposed active sites, attempting to repel Cl^−^ ions while not affecting the exposed active site.^92^ Wang *et al.* synthesised a 3D Ni_3_S_2_/Co_3_S_4_ (NiCoS) nanosheet that was fabricated using a novel one-step hydrothermal method. In 1 M KOH and 0.5 NaCl, as well as 1 M KOH and seawater, the OER performance of the NiCoS electrode is very competitive compared to similar sulphide electrocatalysts. This is likely due to the Co content within the catalyst, requiring overpotentials of 270, 360 and 430 mV to achieve current densities of 100, 500 and 1000 mA cm^−2^, respectively.^98^ In 1 M KOH and seawater, the OER performance declines due to seawater's small particulate and bacterial contaminations (see Table 4). As a result, the catalyst requires overpotentials of 280, 360 and 440 mV to achieve current densities of 10, 100 and 500 mA cm^−2^, respectively.^98^ However, Wang *et al.* used an Ag/AgCl reference electrode during electrochemical testing, which, when exposed to strong alkaline environments, AgCl can become oxidised to Ag_*x*_O, leading to a shift of the reference electrode potential towards the positive direction because of the mixing potential of Ag/Ag_*x*_O and Ag/AgCl interfaces.^99,100^ Using a Hg/HgO reference electrode would have been beneficial. Stability investigations maintain the competitive nature of this catalyst, as over a 100 h chronopotentiometry test (Fig. 9a), the 270 mV overpotential remains constant in simulated seawater and remains stable over the same test in alkaline seawater.^98^

**Table 4:** Sulphide doped OER electrocatalysts and corresponding performance in saline electrolytes

OER catalyst	Ref.	Duration (h)	Electrolyte solution	Cell voltage (V)	Current density (mA cm^−2^)	Overpotentials to achieve current density: *η* (mV)
Ni_3_S_2_/Co_3_S_4_ (NiCoS)	98	>100	1 M KOH + seawater	2.11	100, 500 & 1000	280, 360 & 440
MoS_2_-(FeNi)_9_S_8_/NFF	101	72	1 M KOH + seawater	1.57 + 1.62 respectively	100 + 500	256@100, 329@500
NiFe-LDH-S/CC	95	12	1 M KOH + 0.5 NaCl	1.526	100	296

Thiourea can be utilised as a source of sulphur^102^ as explored by Song *et al.* who synthesised a MoS_2_-(FeNi)_9_S_8_/NFF, further confirming the benefit of S-doping.^101^ Synthesis of the catalyst was carried out using a facile one-step hydrothermal vulcanisation method (Fig. 9b), where heterostructures were grown on a NiFe foam in a solution of Na_2_MoO_4_ and thiourea (an accelerator to vulcanisation), which served as the Mo and S foundation, respectively.^101^ During the hydrothermal reaction, nickel and Fe are reacted with S to create the resulting sulphide. The simple synthesis provides a time-effective method for scaling up electrocatalyst preparation and is beneficial for future studies. The MoS_2_-(FeNi)_9_S_8_/NFF required overpotentials of 238 and 284 mV to achieve current densities of 100 and 500 mA cm^−2^, respectively, in simulated seawater. In alkaline natural seawater, the performance was slightly decreased, with higher overpotentials of 256 and 329 mV at 100 mA cm^−2^ and 500 mA cm^−2^, respectively.^101^ 329 mV at 500 mA cm^−2^ is the best performance observed in this review paper from the S-doped electrocatalyst containing Mo. The better performance can be attributed to the MoS_2_ and (FeNi)_9_S_8_ layer that efficiently controlled the charge distribution, increasing the oxidation of the NiFe site and adsorption of OH intermediates.^101^

Using a different sulphur precursor tends to impact the morphology and crystallinity of the catalyst.^103^ Jung *et al.* investigate using sulphur powder as the precursor for the source of sulphur, synthesising the catalyst by an established hydrothermal process.^95^ The performance of the NiFe-LDH-S350 catalyst (Fig. 9d) displayed an overpotential of 296 mV at 100 mA cm^−2^ in 1 M KOH and 0.5 M NaCl, in comparison to an OER test run on unmodified NiFe-LDH, which reached 100 mA cm^−2^ at an overpotential of 314 mV, illustrating that sulphidation enhances the activity of NiFe-LDH catalyst in a saline electrolyte.^95^ Sulphidation aids the reaction in saline electrolytes, where the negative charge from S^2−^ ions effectively repels the Cl^−^ ions in seawater, decreasing the chloride corrosion, owing to improved stability and performance compared to the original NiFe-LDH catalyst.^95^ However, no detail on the mechanism further deterring OH^−^ ions is given. Stability with the NiFe-LDH-S demonstrated a lower overpotential and 0.7% increase in potential during chronopotentiometry tests over 12 hours, compared to a 2.7% increase for unmodified NiFe-LDH (Fig. 9c).^95^ Further, XPS and TEM analysis was conducted on the sample after the chronopotentiometry test, illustrating that the catalyst morphology was maintained over the tests. XPS of sulphur species observed the presence of M–O–S species post OER and demonstrated the mixed phases of sulphide and hydroxide, reinforcing the hypotheses that sulphur atoms are transformed into sulfoxide species within the matrix of NiFe-LDH, resulting in the excellent catalytic activity and stability of NiFe-LDH-S/CC.^95^

###### 4.4.1.2.2. Sulphate doping

While sulphide doping is a type of anionic doping, sulphate doping (Table 5) is a type of polyanion doping, as SO_4_^2−^ consists of a sulphur atom that is connected to 4 oxygen atoms.^104^ The main difference is that the S^2−^ charge comes from gaining electrons, and the SO_4_^2−^ charge stems from the net charges of each atom.^104^ Studies show that NiS_*x*_ (nickel sulphate) layers act as a sulphur source, generating a polyatomic interface repelling Cl^−^ ions from etching corrosion.^34,54,94^

**Table 5:** Sulphate doping for OER electrocatalyst for DSWE

OER catalyst	Ref.	Duration (h)	Electrolyte solution	Cell voltage (V)	Current density (mA cm^−2^)	Overpotentials to achieve current density: *η* (mV)
Ni_3_S_2_-MoS_2_-Ni_3_S_2_@NF	94	>100	1 M KOH + 0.5NaCl	1.82	100	330
S-NiMoO_4_@NiFe-LDH	105	20	1 M KOH + seawater	1.68 + 1.73	100	315
Mo–Ni_3_S_2_/NF	106	500	1 M KOH + seawater	—	10 + 100	212 + 291 respectively
S-(Ni, Fe)OOH	71	24	Seawater	1.81	500 + 1000	392@500
						462@1000
S-NiFeO_*x*_H_*y*_/CC	107	24	1 M KOH + 0.5 M NaCl	—	100	250
NiFe-NiS_*x*_-NF	23	>1000	1 M KOH + 0.5NaCl	2.1 V + 1.72 V	400 + 1500	300 mV + 380 mV respectively

**Fig. 10:**
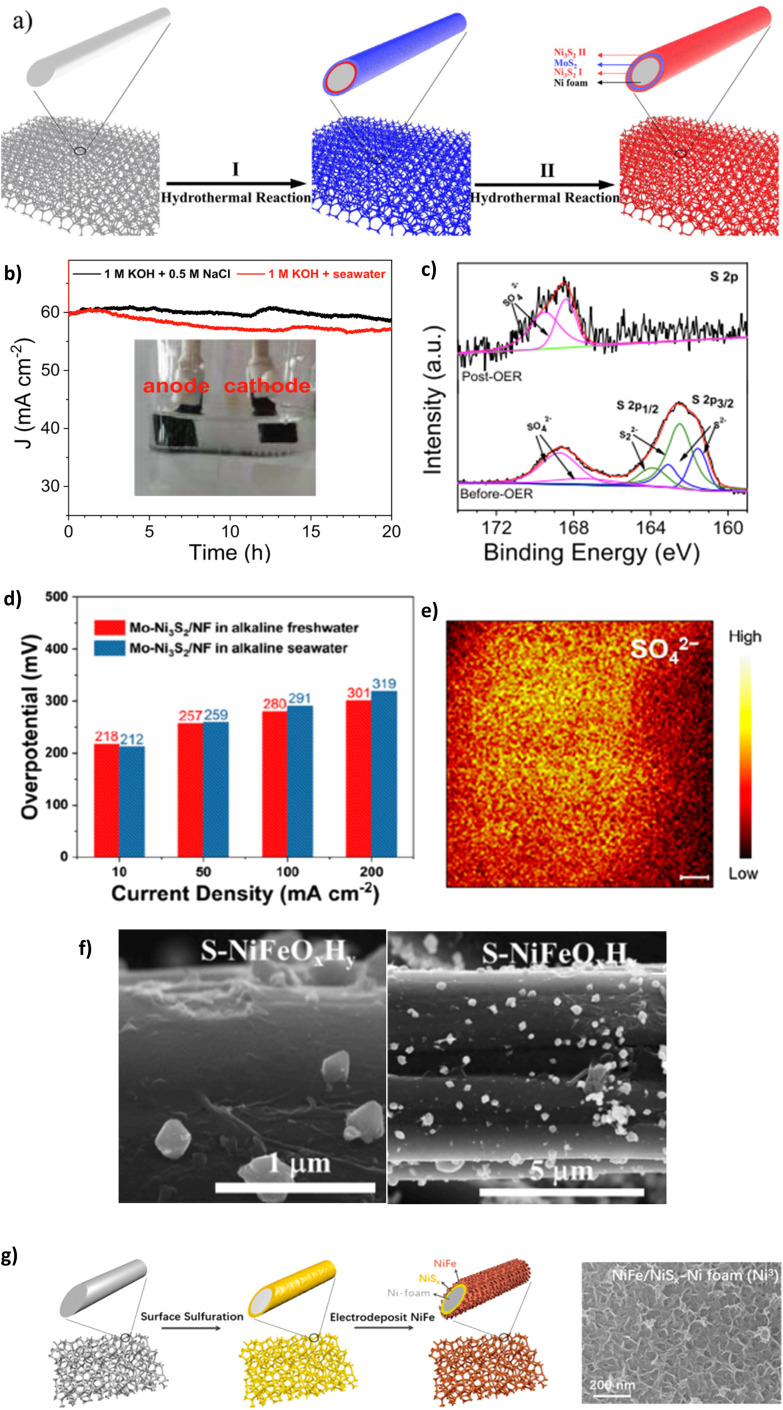
(a) Synthesis schematic of Ni_3_S_2_-MoS_2_-Ni_3_S_2_ using a two-step hydrothermal method, reproduced from ref. 94 with permission from Elsevier, copyright 2021. (b) CP of S-NiMoO_4_@NiFe-LDH at 60 mA cm^−2^, (c) XPS analysis of S-NiMoO_4_@NiFe-LDH post OER, reproduced from ref. 105 with permission from Elsevier, copyright 2022. (d) OER activity of Mo-Ni_3_S_2_/NF in alkaline freshwater and seawater, (e) TOF-SIMS image of Mo-Ni_3_S_2_/NF, reproduced from ref. 106 with permission from American Chemical Society, copyright 2022. (f) SEM images of S-NiFeO_*x*_H_*y*_/CC electrocatalyst after synthesis, reproduced from ref. 107 with permission from Elsevier, copyright 2023. (g) NiFe-NiS_*x*_-NF synthesis process and resulting SEM image of catalyst morphology, reproduced from ref. 23 under Creative Commons Attribution-NonCommercial-NoDerivatives License 4.0 (CC BY-NC-ND).

Highlighting the Cl^−^ repulsion of NiS_*x*_, embedded layer, Li *et al.* prepared a Ni_3_S_2_–MoS_2_–Ni_3_S_2_ on NF as an OER electrode for efficient DSWE.^94^ The electrocatalyst is a mix of nickel sulphide (Ni_3_S_2_) and NiS_*x*_. Since the authors state that the polyanion sulphate layer is responsible for Cl^−^ ion repulsion, it has been included in the sulphate section. The Ni_3_S_2_-MoS_2_-Ni_3_S_2_@NF electrode was synthesised using a two-step hydrothermal process (Fig. 10a). MoS_2_ microspheres evolved on the Ni_3_S_2_ surface by decomposition of (NH_4_)_2_MoS_4_ using a hydrazine hydrate (HZH) reduction reaction.^94^ The MoS_2_ layer provides beneficial metallic properties and abundant active sites coupled with NiS_*x*_, which boosts electron transfer and improves water-splitting efficiency.^94^ A subsequent hydrothermal process anchors Ni_3_S_2_ nanoparticles onto the MoS_2_ coating.^94^ This is a sandwich Ni_3_S_2_ layer that provides chloride corrosion protection on the exterior and interior of the electrode. In 1 M KOH and 0.5 M NaCl (pH 14) at room temperature, chronopotentiometry was carried out over a 50 h period at a current density of 100 mA cm^−2^, which induced a modest overpotential of 330 mV. Over 50 h, the catalyst remained stable, with a negligible increase in overpotential (330 mV to 331 mV), demonstrating a 0.02 mV h^−1^ degradation rate, the lowest observed in the literature covered in this review, confirming the OER stability of the sulphide-rich NiS_*x*_ sandwich layers on Ni_3_S_2_-MoS_2_-Ni_3_S_2_@NF in simulated seawater.^94^ The NiS_*x*_ layers repelled Cl^−^ within seawater from the surface of the electrode. With the stability achieved, it would have been beneficial to increase the duration of the experiment to explore whether it remains consistent and comparable to similar studies.

Doping NiMo catalysts with polyanions such as sulphate to prevent chloride corrosion while benefiting from a reduced energy barrier to the OER is a desirable combination and has thus been explored by a few authors.^105,106,108^ To this end, Wang *et al.* synthesised 3D core–shell nanostructures incorporating a crystalline and amorphous NiFe-LDH that is placed on sulphur-doped NiMoO_4_ nanorods supported on a NF substrate.^105^ The electrocatalyst is synthesised using a time-consuming three-step process consisting of hydrothermal, vulcanisation and electrodeposition techniques. When tested in simulated seawater and natural seawater at 100 mA cm^−2^, the overpotential was 273 mV and 315 mV, respectively. This decline in performance is attributable to the bacteria and microbes present in raw seawater, fouling electrodes and poisoning catalysts.^105^ Post OER XPS (Fig. 10c) analysis reveals that the peak of the metal–S bond disappears and the peak intensity for SO_4_^2−^ increases substantially, indicating surface reconstruction and thus, the corrosion resistance can be attributed to the multivalent sulfate ions.^105^ The study also uses a substantial *IR* compensation of 90%, which can significantly inflate the reported performance. The study argues that the incorporated SO_4_^2−^ ions repel Cl^−^ ions present in seawater; however, the effectiveness of the catalyst to withstand a simulated seawater electrolyte was limited, as proven in a chronoamperometry test conducted in 1 M KOH + 0.5 M NaCl reveals the instability of the catalyst, where at 60 mA cm^−2^ even over a relatively short period of 20 h, the current density attenuation rate is 3% and increases to 5% in 1 M KOH and seawater. However, the reason why this occurs is not given. It is likely the catalyst begins to shed off the substrate, as seen as the darker layer within the cell setup (Fig. 10b), and it appears not all the electrode is submerged in the electrolyte, meaning not all the surface area is used in the reaction and over the stability test duration the concentration of the electrolyte will change as OH^−^ are evolved into O_2_. Critically, the reduction in stability with the catalyst is likely due to the cell setup rather than the activity of the catalyst; similar electrocatalysts^94^ present significantly better stability without being vastly different in metals used, demonstrating that the structure and organisation of catalyst layers in the electrode are fundamental. Retaining the attachment of various metals and compounds to substrates and supports becomes progressively challenging.^109^ This difficulty is compounded at higher current densities where gas evolution is more intense, increasing the likelihood of catalyst shedding or peel-off. Using binders such as Nafion ionomers, anion exchange ionomers, and non-ionic PTFE binders can aid catalyst adhesion to the substrate.^109^ Nafion acts as an effective binder and further improves the interfacial interaction between electrolyte and catalyst, improving stability and performance by up to 20% compared to Nafion-free catalyst layers.^110^

Lan *et al.* synthesised a Mo-doped Ni_3_S_2_ nanocluster array applied to NF (Mo–Ni_3_S_2_/NF), where the catalyst is prepared using a single-step modified solvothermal methodology using thiourea as the sulphur source at 160 °C for 6 hours.^106^ The introduction of Mo progresses the nickel and S organisation, improving electronic interactions and increasing OER reaction kinetics and long-term stability, according to the authors.^106^ The performance tests for the catalyst were carried out in both 1 M KOH and seawater (collected from Shenzhen, China), with overpotentials of 212 mV at 10 mA cm^−2^ and 291 mV at 100 mA cm^−2^.^106^ Impressively, the performance in alkaline seawater is almost identical to that in alkaline freshwater at modest current densities (Fig. 10d), demonstrating the chloride repellence of the S ions at the electrode surface. Furthermore, the remarkable stability of the Mo–Ni_3_S_2_/NF can be observed for >500 h at 100 mA cm^−2^ but uses *IR* compensation without stating that value nor the overpotential increase. The stability can be attributed to the presence of residual sulphate polyanions on the surface of the catalyst, as illustrated using time-of-flight secondary-ion mass spectrometry (TOF-SIMS) (Fig. 10e). This study demonstrates the benefit of sulphate doping on stability and Mo doping brings to the OER activity; further research could benefit from building upon this work to improve the ability to achieve high current densities.

Modification to electrocatalysts typically involves adopting a subsequent synthesis step or increasing the complexity of synthesis. As a result, increasing the efficiency of catalyst synthesis is vital when creating an easy electrocatalyst to scale up. Yu *et al.* synthesised a highly porous S-doped NiFe (oxy)hydroxide (S-(Ni, Fe)OOH) *via* a more efficient approach than existing electrodeposition techniques that tend to result in weak contact between the catalyst and substrate.^71^ NF is immersed and reacted with a solution of Fe(NO)_3_·9H_2_O and sodium thiosulfate (Na_2_S_2_O_3_·5H_2_O) and instantly etched to produce the highly porous NiFe oxy-hydroxide layer.^71^ Immersing the substrate in a precursor solution is not a convincing method to improve adhesion between substrate and catalyst. The authors argue that good contact between the catalyst and substrate is created. Still, at higher current densities, rapid gas diffusion will occur and put stress on the catalyst and substrate contact. The high porosity, hydrophilic features and large surface area result in remarkable catalytic performance within a seawater electrolyte; the performance is 300, 398 and 462 mV at current densities of 100, 500 and 1000 mA cm^−2^, respectively. At 462 mV, the overpotential is very close to entering a region where the ClOR can theoretically evolve. Stability tests were conducted for 100 h at a current density of 100 mA cm^−2^ and 500 mA cm^−2^ in varying electrolytes. The stability will be due to the sulphur groups present on the catalyst surface, which are in the form of thiosulphate and sulphate stemming from the oxidation of Na_2_S_2_O_3_ during the reaction; this is confirmed by Fourier-transform infrared spectroscopy (FTIR).^71^ A 0.7 mV h^−1^ degradation rate is seen at 500 mA cm^−2^ and a 0.5 mV h^−1^ degradation rate at 100 mA cm^−2^ in 1 M KOH + 1 M NaCl, which is high but still better than many earth-abundant OER electrocatalysts reported in the literature^65,71,91^ but lacking behind electrocatalysts containing metals of high economic value in this review.

Using sodium thiosulfate as a sulphate source is further explored by Zhang *et al.* who synthesised an S-doped NiFe oxide/hydroxide with a CC substrate, denoted S-NiFeO_*x*_H_*y*_/CC.^107^ The electrocatalyst was synthesised using a two-step electrodeposition and hydrothermal method.^107^ SEM images (Fig. 10f) reveal that the S-doped layer was sparsely distributed across the surface and loosely bonded to the electrode surface, which puts doubt on the Cl^−^ repellency of the coating due to a large surface area of active sites exposed to Cl^−^ ions but may explain why the catalyst can achieve lower overpotentials than other S-doped layer catalysts since the inherent negative charge can also repel OH^−^. In 1 M KOH and 0.5 M NaCl at current densities of 10, 100 and 500 mA cm^−2^, the overpotentials are 265, 331 and 409 mV, respectively; in comparison to the other reported S-doped oxides and hydroxides, these overpotentials are competitive.^71,95,107^

The use of sulphur powder was initially explored in the sulphide section but is further used by Kuang *et al.* who report a polyanion sulphate and carbonate passivated NiFe, NiS_*x*_, NF core anode (Ni^3^) (Fig. 10g), demonstrating increased activity and corrosion resistance in an alkaline electrolyte containing chloride.^23^ The anode comprises negatively charged polyanions produced from the constant current activation of the NiS layer. This involves the oxidation of the NiS layer, causing anodic etching and leading to the formation of sulphate ions that subsequently migrated to the NiFe layer, intercalating with the carbonate ions known to exist in the KOH solution. As a result, the fundamental nickel sulphate layer is created, which repels Cl^−^ anions that occur in seawater, creating corrosion resistance.^23^ The authors argue that a polyatomic anion layer beneath the main catalyst layer inhibits chloride corrosion by enabling the reactant to diffuse into the bulk solution once created at the catalyst interface. However, this doesn't explain the ion selectivity of the catalyst; there is no mechanism establishing whether the catalyst solely attracts OH^−^ ions over Cl^−^ ions. Increasing electron density around the catalyst layer hinders further OH^−^ adsorption and thus O_2_ gas evolution, meaning a high activation energy is required to overcome the O–O coupling thermodynamic barrier.^23,77^ According to the research, the catalyst can achieve a 380 mV overpotential at 1.5 A cm^−2^ in 1 M KOH + 0.5 M NaCl. However, it is mentioned that a 95% *iR* compensation is used, which likely overcompensated the results, particularly at high current densities (>1 A cm^−2^), enough to change a ‘mediocre’ catalyst into a ‘promising’ catalyst, typically an *iR* compensation in the region 80–85% is reasonable.^111^ The electrolyser could achieve a current density of 400 mA cm^−2^ with a cell potential of only 2.1 V under natural seawater conditions with 1 M KOH added to seawater at room temperature. The electrolyser only required a potential of 1.72 V at industrial electrolysis conditions at 80 °C.^23^ The paper further identified that it was possible to maintain current density levels of 400, 800 and 1000 mA cm^−2^ in the system for 500 hours in 1 M KOH and 0.5 M NaCl.^23^ Critically, while the high *iR* compensation is not excellent practice from the authors, Kuang *et al.* have synthesised a very stable and durable OER electrocatalyst, showing real promise for seawater electrolysis; the performance is still achieved in a natural seawater environment. Not only that, but the duration of the test also simulates a real-world exposure (1000 hours = 41.66 days), the longest of any catalyst tested in this review.

###### 4.4.1.2.3. Phosphide doping

Phosphide (P^3-^) doping has gained attention for modifying OER electrocatalysts due to high intrinsic catalytic activity, tuneable composition, and structure.^91,112^ Wu *et al.* presented a bimetallic ternary phosphide heterostructured Ni_2_P-Fe_2_P electrocatalyst that incorporated a nanosheet morphology on an NF substrate using phosphidation (Table 6).^91^

**Table 6:** Phosphide doping for OER electrocatalyst for DSWE

OER catalyst	Ref.	Duration (h)	Electrolyte solution	Cell voltage (V)	Current density (mA cm^−2^)	Overpotentials to achieve current density: *η* (mV)
Ni_2_P-Fe_2_P	91	36@100	1 M KOH + seawater	1.811@100 + 2.004@500	100 + 1000	305@100
		23@500				431@1000

**Fig. 11:**
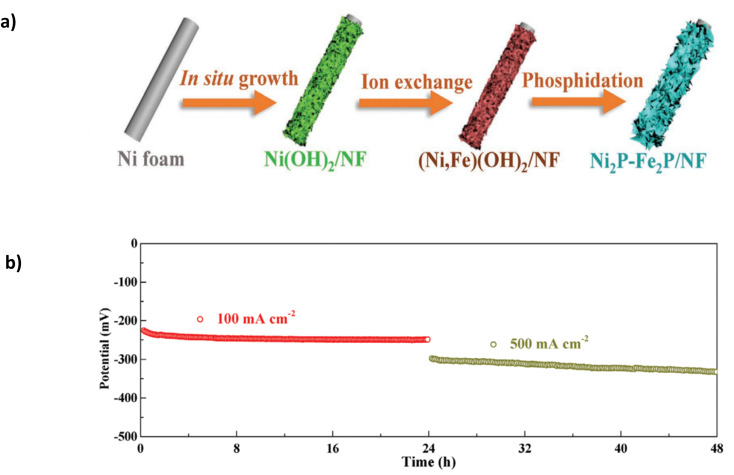
(a) Ni_2_P-Fe_2_P/NF three-step synthesis method. (b) Stability of Ni_2_P-Fe_2_P/NF in 1 KOH + seawater at 100 mA cm^−2^ and 500 mA cm^−2^. Reproduced from ref. 91 with permission from John Wiley and Sons, copyright 2020.

The catalyst was synthesised using a three-step approach (Fig. 11a) involving multiple immersion steps. Firstly, a facile “etching growth” method by which NF is immersed in 3 M HCl and DI water, creating uniform nanosheets which change the sample's wettability to hydrophobic, allowing more Fe cations to load onto the catalyst. The substrate was immersed in an iron nitrate solution to initiate ion exchange with the Fe cations, creating (Ni, Fe)(OH)_2_. A final phosphidation process creates Ni_2_P-Fe_2_P/NF.^91^ Critically, the immersion process will likely lead to a weak bond between the catalyst and substrate without artificial binders. As the catalyst is subjected to higher current densities with vigorous bubble formation, it may strain the catalyst, affecting stability. In 1 M KOH and seawater, it exhibited overpotentials of 305 mV and 431 mV at current densities of 100 mA cm^−2^ and 1000 mA cm^−2^, respectively.^91^ The performance can be attributed to the synergistic effect of the binary components (Ni and Fe). Furthermore, the nanosheet construction and hydrophilic feature aid the diffusion of the electrolyte and improve the discharge of gases.^91^ Good stability is observed at 100 mA cm^−2^ for a continuous 36 hours and subsequent 23 hours at 500 mA cm^−2^ (Fig. 11b). At 500 mA cm^−2^, the potential gradually increases by approximately 50 mV, indicating a high degradation rate of 2.17 mV h^−1^ and eventually leading to complete deterioration of the catalyst. Critically, the study lacks the performance and stability attributes observed in similarly reported catalysts for DSWE.

###### 4.4.1.2.4. Phosphate dopants

Phosphate (PO_4_^3−^) doping, particularly with nickel and Fe, has seen a growth in published papers over the past years due to their ability to improve electrocatalytic reactions as well as their ability to inhibit chloride corrosion as a polyanion layer (Table 7).^113–115^ Song *et al.*^93^ investigated this and synthesised a Mo-doped OER catalyst, using phosphate-doping to create Mo-NiFe-PO_3_/NFF using a two-step hydrothermal and annealing approach. The nickel-iron foam (NFF) is etched in place *via* chemical oxidation in a solution of Na_2_MoO_4_ and H_2_O_2,_ creating a hollow ‘bird nest’ structure, where sheets of Mo-doped NiFe hydroxide act as the wall.

**Table 7:** Phosphate-doped OER electrocatalysts for DSWE

OER catalyst	Ref.	Duration (h)	Electrolyte solution	Cell voltage (V)	Current density (mA cm^−2^)	Overpotentials to achieve current density: *η* (mV)
Mo-NiFe-PO_3_/NFF	93	100	1 M KOH + seawater	1.65 + 1.78 respectively	100, 500 + 1000	263@100
						311@500
						356@1000
S-NiFe-Pi/NFF	116	100	1 M KOH + seawater	1.68 @ 100 + 1.8 @ 500	100 + 500	241@100
						295@500
						325@1000

**Fig. 12:**
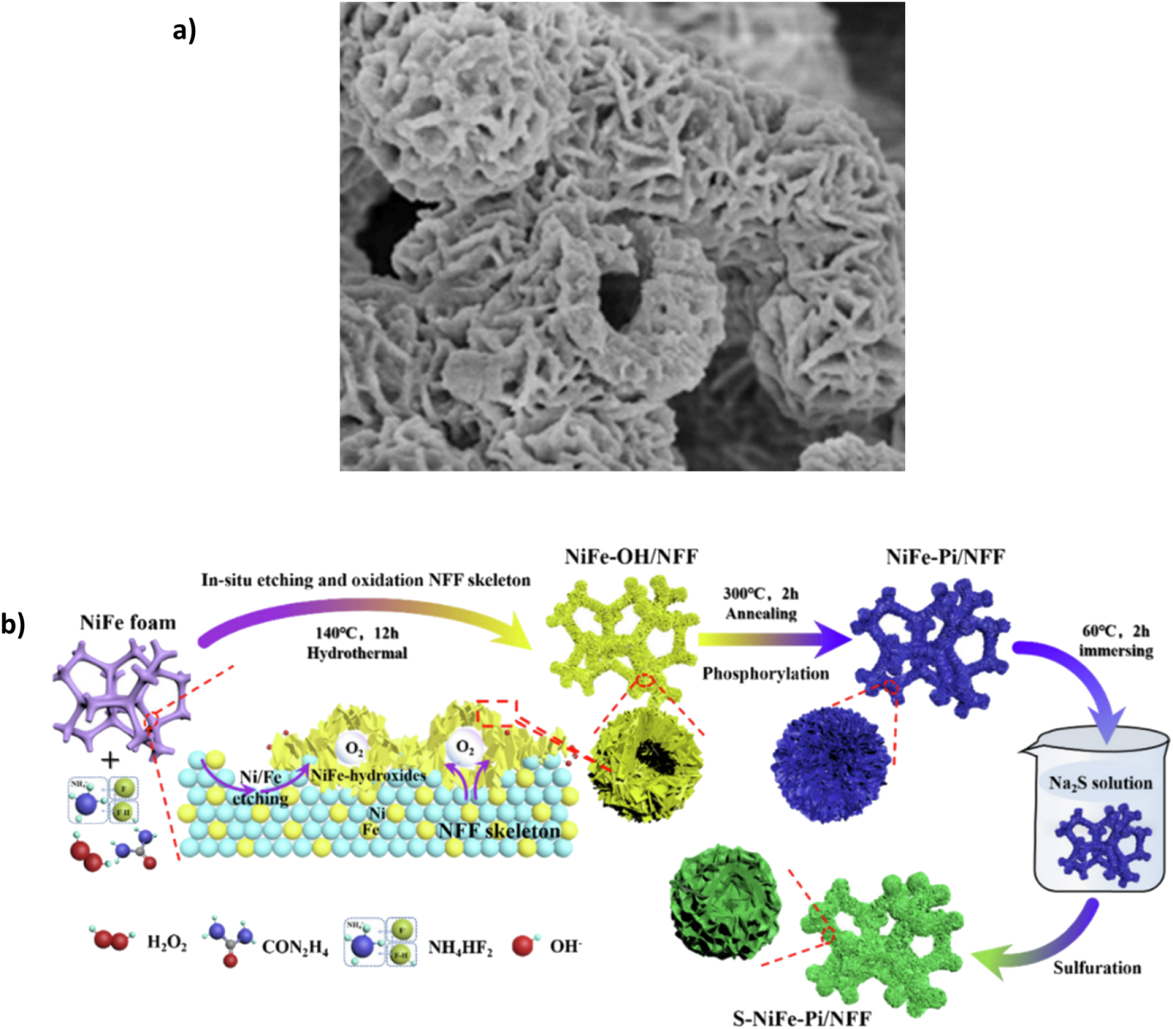
(a) Mo-NiFe-PO_3_/NFF under SEM magnification, Reproduced from ref. 93 with permission from Elsevier, copyright 2022. (b) S-NiFe-Pi/NFF three-step synthesis procedure, reproduced from ref. 116 with permission from Elsevier, copyright 2023.

Annealing phosphorylation transforms the hydroxide into Mo-doped NiFe phosphate. SEM images reveal the catalyst's consistent distribution and good adhesion on the substrate (Fig. 12a). Mo provides high corrosion resistance due to the link with phosphate polyanions, which resist Cl^−^ ions on the surface in seawater.^93^ In 1 M KOH + 0.5 M NaCl, the electrocatalyst can achieve current densities of 100 mA cm^−2^ and 500 mA cm^−2^ with overpotentials of 247 and 294 mV, respectively. In 1 M KOH + seawater, the overpotentials rise to 263 and 311 mV for the same current densities. Surprisingly, it reaches 1 A cm^−2^ with an overpotential of 356 mV, which is impressive and only 53 mV higher than Ag/NiFe-LDH.^80^ The increase in potential when using natural seawater is attributed to the fact that the small particles and bacterial contaminations in seawater can block active sites and contaminate the catalyst.^93^ While the overpotentials are highly competitive with other reported electrocatalysts, stability is limited. The paper highlights a 100 h chronoamperometry test in 1 M KOH + seawater at approx. 100 mA cm^−2^, the current density fluctuates throughout the test and maintains 93.4% of the original level, a 6.6% decline over a 100 h period, which highlights some issues for further stability improvement.

Song *et al.* recently synthesised a combination of a polyanion and anionic doping, namely an S-modified NiFe phosphate (Pi) on NiFe foam (NFF). Synthesis of the S-NiFe-Pi/NFF uses a three-step approach (Fig. 12b), incorporating an *in situ* oxidation-phosphorylation-anion regulation process.^116^ Interestingly, the authors add the sulphur layer as the primary layer to repel Cl^−^ ions. The sulphur layer replaces the phosphate ion (PO_4_^3−^), becoming the dominant anion-repellent layer.^116^ The introduction of the S^2−^ layer has been explored by a few works. However, no study describes an in-depth mechanism behind this, specifically the need for ion selectivity from the electrode surface. In 1 M KOH + 0.5 M NaCl, the electrode could achieve 100 mA cm^−2^ and 500 mA cm^−2^ with overpotentials of 232 mV and 278 mV, respectively. In 1 M KOH + seawater, the same current densities can be achieved with overpotentials of 241 mV at 100 mA cm^−2^ and 295 mV at 500 mA cm^−2^. Furthermore, the catalyst can reach 1 A cm^−2^ with an overpotential of 325 mV.^116^ Interestingly, the paper also investigates the performance of the catalyst without the S layer, designated NiFe-Pi/NFF; in 1 M KOH at 100 mA cm^−2^, NiFe-Pi/NFF required an overpotential of 246 mV, which is higher than that of S-NiFe-Pi/NFF (232 mV).^116^ The S layer distorts the NiFe-phosphate lattice and improves the adsorption capability of intermediates, advancing OER electrocatalytic activity.^116^ Over a 100 h period using chronopotentiometry at 500 mA cm^−2^ in 1 M KOH + seawater, the potential was held at 1.525 V *vs.* RHE, and the test showed that 86.6% of the original current density was maintained, meaning the current density dropped to approx. 433 mA cm^−2^, which is approximately a degradation rate of 0.67 mA h^−1^.^116^

###### 4.4.1.2.5. Other dopants

Some less frequently used dopants are shown in Table 8. Specifically, nitride doping, a type of anionic doping and borate doping, a type of polyanion doping, both dopants aim to incorporate a repulsion layer that repels Cl^−^ ions.

**Table 8:** Other anionic and polyanionic dopants for OER electrocatalyst for DSWE

Dopant	OER catalyst	Ref.	Duration (h)	Electrolyte solution	Cell voltage (V)	Current density (mA cm^−2^)	Overpotentials to achieve current density: *η* (mV)
Nitride	NiMoN@NiFeN	51	100	1 M KOH + 0.5 NaCl	—	500	347
Borate	NiFeB_*x*_	96	100	1 M KOH + 0.5 NaCl	—	100, 500 + 1000	328, 400 + 470

The addition of nitride doping can result in exceptional corrosion resistance, improved conductivity and stability.^29,117,118^ Nitrogen atoms alter the d-band density states, providing greater catalytic activity than metal materials.^29,119^ Yu *et al.* synthesised a NiMoN@NiFeN catalyst in a three-dimensional core–shell composition with extensive surface area and high-density active sites.^51^ The synthesis provides an excellent example of how to mitigate catalyst shedding since the structure of the catalyst is a region of conductive NiMoN nanorods grown on an NF substrate with a layer of NiFeN nanoparticles uniformly deposited on top, ensuring effective charge transfer.^51^ This is an example of a self-supported catalyst; self-supported electrodes offer several advantages, including a more straightforward preparation process, lower cost, abundant catalytic sites, rapid charge transfer, and the avoidance of electrocatalyst shedding.^109^ This is achieved through the direct *in situ* growth of catalytic material on conductive substrates such as carbon cloth or NF or by using an oriented solid-phase synthesis (OSPS) method to grow the material vertically on the substrate. These features make self-supported electrodes optimal for boosting catalytic activity and ensuring long-term stability at high current densities.^109^ The catalyst showed excellent performance, including an overpotential of 369 mV at 500 mA cm^−2^ in 1 M KOH and natural seawater, achieving 347 mV at 500 mA cm^−2^ in 1 M KOH + 0.5 M NaCl and 410 mV at 1 A cm^−2^.^51^ This performance can be attributed to the stable structure, hydrophilic surface and high conductivity of NiMoN nanorods that are uniformly decorated with NiFeN nanoparticles, ensuring fast and efficient charge transfer.^51^ At 100 mA cm^−2^ for 100 h at room temperature, the current density decreases by 3.82% from 500 mA cm^−2^ to 480.9 mA cm^−2^ due to strong bubble adsorption on the catalyst surface, blocking active sites. The outer NiFeN layer evolved amorphous layers of NiFeOOH and NiFeO_*x*_ during the OER process.^51^ As a result, NiFeOOH and NiFeO_*x*_ mitigate the adsorption of Cl^−^ ions from the catalyst surface and aid the conversion of OH^−^ to O_2_.^51^

**Fig. 13:**
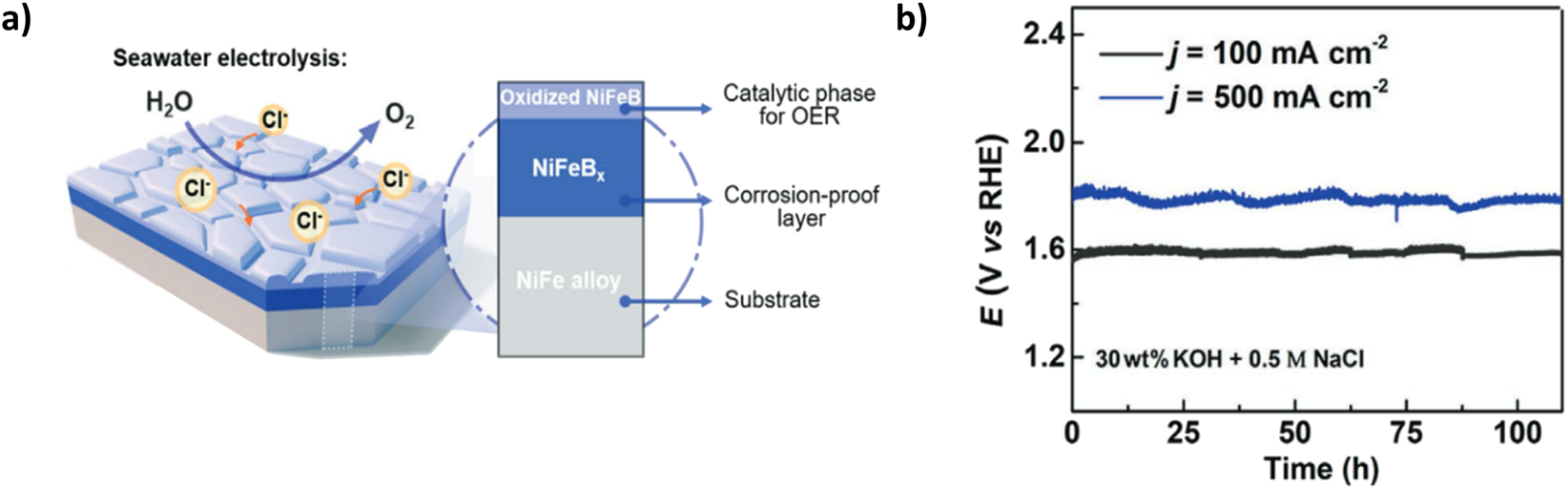
(a) Schematic of NiFeB_*x*_ electrode with a breakdown of relevant layers (b) chronopotentiometry curve of NiFeB_*x*_ electrode in simulated seawater at 100 and 500 mA cm^−2^. Reproduced from ref. 96 with permission from John Wiley and Sons, copyright 2021.

Borate doping offers promise as another polyanion inter/outer layer for seawater electrolysis. Li *et al.* present a three-tier NiFe electrode with a conductive oxidised NiFeB_*x*_ outer layer NiFeB_*x*_ interlayer on a NiFe substrate.^96^ The catalyst was synthesised using thermal boronization with boron powder and a subsequent electrochemical oxidation process using cyclic voltammetry to create the oxidised outer catalyst layer (Fig. 13a).^96^ The NiFeB_*x*_ interlayer improves the corrosion resistance, and the oxidation process of the NiFeB_*x*_ outer layer initiates a highly active phase of γ-(Ni, Fe)OOH.^96^ The higher oxidation state of the nickel created a metaborate (borate anion that has been oxidised (BO_2_)) involvement, improving the oxidation state of Ni, Fe(OH)_2_ to γ-(Ni, Fe)OOH through the OER process by finely tuning the electronic structure of nickel sites, allowing an increase in surface oxygen adsorption.^96^ This equates to the high catalytic performance of the electrode, which achieves current densities of 100, 500 and 1000 mA cm^−2^ in 1 M KOH & 0.5 M NaCl with corresponding overpotentials of 328, 400 and 470 mV, respectively.^96^ However, at 500 and 1000 mA cm^−2^, the extra energy required to overcome the NiFeB_*x*_ layer becomes apparent from the overpotential at 400 and 470 mV and is only 10 mV away from the ClOR theoretical potential region. Chronopotentiometry tests reveal good stability at 100 mA cm^−2^ for over 100 hours (Fig. 13b), but at 500 mA cm^−2^, bubble formation is an issue but not significant enough to cause the catalyst to peel off the substrate. The slight current peaks and troughs indicate the blocking of active sites by the formed bubbles. The paper provides excellent insight into incorporating boride layers into OER catalysts to repel Cl^−^ ions. Still, a lack of insight into using natural seawater is a limitation, and the increased overpotential at high current densities compared to other polyanion doping methods is not competitive.

#### 4.4.2. Outer layer protection

**Fig. 14:**
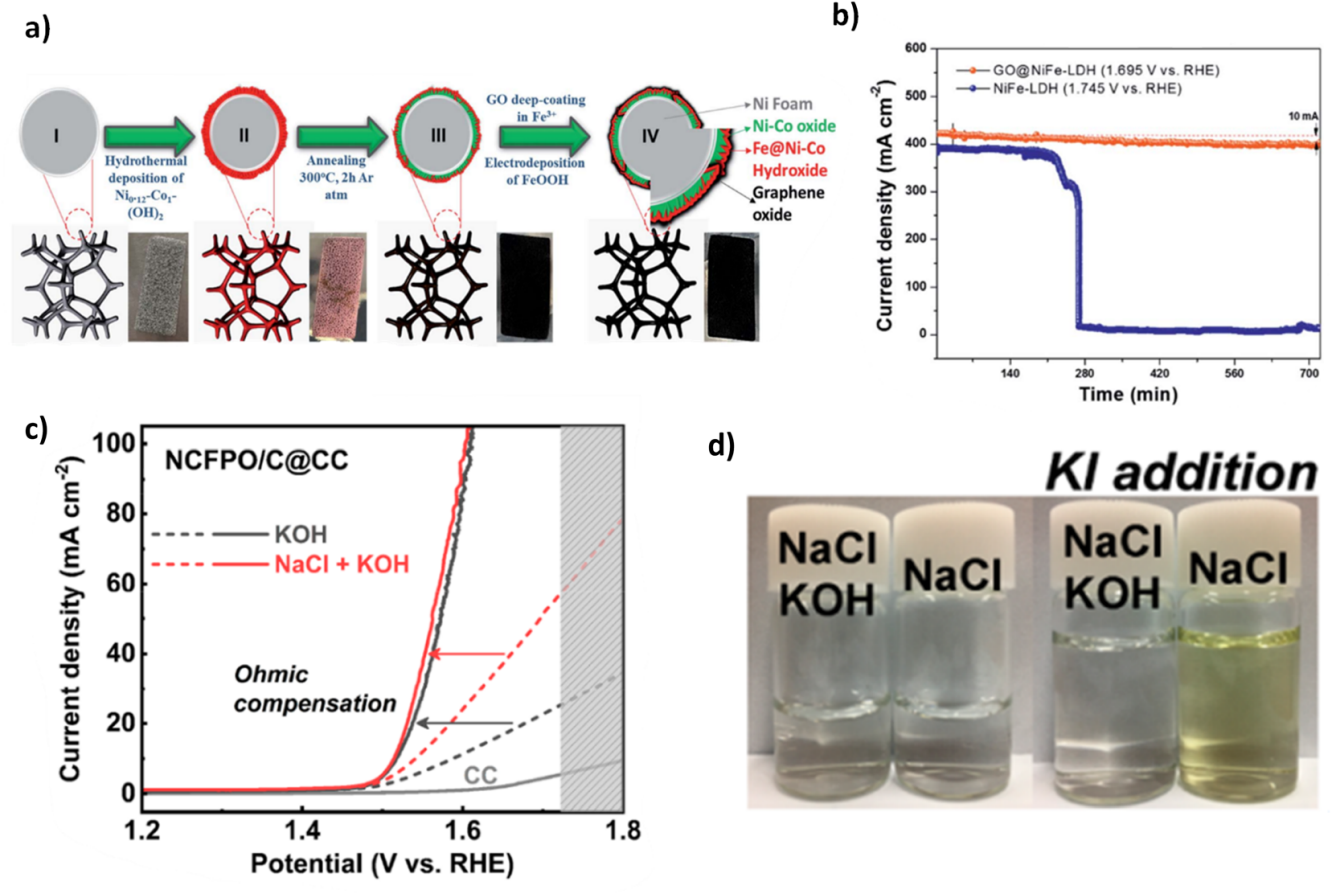
(a) Synthesis steps for GO@Fe@Ni–Co@NF, (b) chronopotentiometry test of GO@Fe@Ni–Co@NF, reproduced from ref. 121 with permission from Royal Society of Chemistry, copyright 2020. (c) Polarisation curves of NCFPO/C@CC in KOH and NaCl containing electrolyte, (d) colour change shown in both a solution of KOH and KOH & NaCl when adding KI, adapted from ref. 120 with permission from American Chemical Society, copyright 2020.

A simple technique to enhance the corrosion resistance to Cl^−^ ions is to introduce a protective outer layer to an existing highly active OER electrocatalyst; some work (shown in Table 9) has identified using carbon or graphene as an outer layer is effective (Fig. 14). Jadhav *et al.* present a Graphene oxide (GO) FeOOH deposited on β-phase Ni-Co hydroxide, denoted as GO@Fe@Ni–Co@NF. GO@Fe@Ni–Co@NF has an intricate structure in that FeOOH is deposited on β-Ni–Co, serving as the active and stable OER catalyst, while the GO outer layer is used to enhance the corrosion resistance.^121^ GO was selected as a protective layer to mitigate Cl^−^ corrosion, as GO membrane is well established in the reverse osmosis (RO) desalination process and is proven to allow effective diffusion of gases.^121–126^ The electrocatalyst is synthesised using a three-step hydrothermal, annealing and electrodeposition process (Fig. 14a). This complex and time-consuming synthesis, compared to similar performing electrocatalysts, means regardless of performance, it is challenging to scale up, limiting further applications. Notably, the use of Ni–Co LDH in this catalyst was created during the hydrothermal step, where it is classed as a β-phase. β-phase metal hydroxides are chosen here due to smaller interlayer spacing (<4.74 Å) than α-phase metal hydroxides (>8 Å). A smaller interlayer spacing makes for greater chloride corrosion resistance in the catalyst due to the inability of Cl^−^ ions to intercalate during water oxidation.^121,127^ The multi-layered three-dimensional electrode could achieve a current density of 1000 mA cm^−2^ at an overpotential of 345 mV (with *iR* compensation). The stability can be attributed to the GO coating on the catalyst surface and the use of β-phase Ni–Co LDH (Fig. 14b). compares the synthesised catalyst to a β-NiFe-LDH, which lasts approximately—280 hours (more competitive than most reported nickel electrocatalysts in literature). The GO aids in preventing chloride corrosion, and the catalyst can be further used for 378 h (*i.e.* 15.75 days) at a current density of 1000 mA cm^−2^ with a negligible decrease in catalytic activity (10 mA reduction over the period).^121^

**Table 9:** OER electrocatalysts with outer layer protection and corresponding performance in a saline electrolyte

OER catalyst	Ref.	Duration (h)	Electrolyte solution	Cell voltage (V)	Current density (mA cm^−2^)	Overpotentials to achieve current density: *η* (mV)
GO@Fe@Ni–Co@NF	121	378	1 M KOH + seawater	—	500	303
NCFPO/C@CC	120	100	0.5 M NaCl + 0.1 M KOH	1.6	100	—

In addition to a GO outer layer, Song *et al.* investigated using a carbon outer layer to improve corrosion resistance.^120^ He *et al.* created a carbon-coated cobalt sodium pyrophosphate catalyst on a carbon cloth substrate, written as Na_2_Co_1−*x*_Fe_*x*_P_2_O_7_/C on CC but denoted as NCFPO/C@CC. Synthesis is particularly complex using a 4-step process of sol–gel method, initial heat treatment, ball milling and a secondary heat treatment, one of the more complex synthesis techniques seen in this review. The electrocatalyst was tested in a solution of 0.5 M NaCl and 0.1 M KOH, a significantly lower buffer concentration than used in other studies to investigate the OER activity. A cell setup could achieve 100 mA cm^−2^ at 1.6 V (Fig. 14c), where the NaCl increased the ionic conductivity, resulting in an earlier onset OER potential and illustrating that Cl^−^ did not influence the performance. The OER performance stems predominantly from the OH^−^ evolved on the surface of NCFPO/C@CC acting as the active sites, improving catalytic activity for the OER. To prove whether Cl^−^ evolved, the authors performed iodide titration (Fig. 14d) to determine whether chlorine had actively evolved in the solution. A colour change was observed in a pure NaCl electrolyte at low current densities but not in 0.5 M NaCl and 0.1 M KOH (Fig. 14d).^120^ This illustrated that the ClOR did not occur in the active alkaline saline solution and is owed to the outer carbon layer coated on the electrode surface, which is further proved in a 100 h chronopotentiometry test where no increase in potential is observed. While the synthesis is particularly complex, it could be simplified using another OER electrocatalyst in future work. The carbon-coated outer layer demonstrated effective Cl^−^ resistance; however, the effect of carbon oxidation of this layer is not studied here, likely due to the low current densities. However, at elevated anodic potentials, carbon corrosion could occur.^67^

#### 4.4.3. Ion selectivity

The intricate chemistry behind the selective adsorption of anions onto an electrode surface has only recently been explored by a few authors (Table 10).^128,129^ A benefit of selecting specific ions to the electrode surface is mitigating the need for strong alkali addition (KOH) to widen the operating region for the OER, as theoretically only OH^−^ will be adsorbed onto the electrode; this results in reduced costs for the setup ($800 t^−1^ for KOH^14^).

**Table 10:** OER electrocatalysts with an ion-selective layer and corresponding performance in saline electrolytes

OER catalyst	Ref.	Duration (h)	Electrolyte solution	Cell voltage (V)	Current density (mA cm^−2^)	Overpotentials to achieve current density: *η* (mV)	
NiFe-LDH/NF	129	12	1 M KOH + 0.5 NaCl	1.6	100 + 500		227 + 257
Cr_2_O_3_–CoO_*x*_	128	100	Unbuffered seawater	1.87	1000		—
SiO_*x*_/Pt	130	12	0.5 M KHSO_4_ + 0.6 M KCl	1.90	160		—
MnO_2_/CC	131		Unbuffered seawater	∼2.25	100		1098

**Fig. 15:**
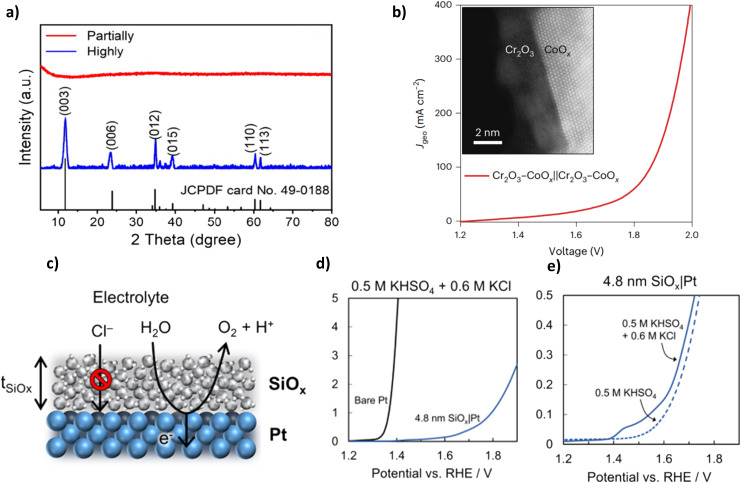
(a) XRD pattern showing the difference between a highly crystalline NiFe-LDH and an amorphous NiFe-LDH, reproduced from ref. 129 with permission from American Chemical Society, copyright 2021. (b) Overall water splitting performance of Cr_2_O_3_–CoO_*x*_ in natural seawater, with an inset image of high-angle annular-dark-field-scanning transition electron microscopic (HAADF-STEM) image, reproduced from ref. 128 with permission from Springer Nature, copyright 2023 (c) SiO_*x*_/Pt electrode schematic selectively blocking Cl^−^ ions and allowing OH– to evolve into O_2_, (d) LSV curve of SiO_*x*_/Pt electrode in 0.5 M KHSO_4_ + 0.6 M KCl at scan rate of 20 mV s^−1^, pH 0.8 and 25 °C, (e) LSV curve of SiO_*x*_/Pt electrode in 0.5 M KHSO_4_ + 0.6 M KCl and 0.5 M KHSO_4_ at scan rate of 20 mV s^−1^, pH 0.8 and 25 °C, reproduced from ref. 130 with permission from American Chemical Society, copyright 2021.

The selectivity phenomenon is explained by Pearson's hard-soft acid-base principle (HSAB), which states that harder bases attract harder acids, and the same is true for softer bases and softer acids.^129,132^ Acids (metal ions) function as electron pair acceptors, and bases are ligands that serve as electron pair donors. Metal ions with high positive charges and small ionic sizes tend to be hard acids. The hardness of an acid can be defined by the p*K*_a_ value, which determines the strength of an acid from the acid dissociation constant (how tightly a Brønsted acid holds a proton).^133^ Tu *et al.* demonstrated this concept using two different NiFe-LDHs, one of a highly crystalline structure and one of a partially crystalline nature (Fig. 15a).^129^ The partially crystalline sample has an amorphous phase intercalated with nanometer-sized facets. The study investigates the varying adsorption behaviours of OH^−^ and Cl^−^ and the influence of crystallinity on these mechanisms. The catalyst was synthesised using a commonly used hydrothermal method set out in literature (immersing NF in 0.50 mM of Ni(NO_3_)_2_, 0.50 mM of Fe(NO_3_)_3_, and 5.00 mM of urea and heated to 120 °C for 12 h). The main difference between synthesis is that the partially crystalline used metal chloride precursors are added under intense stirring instead of an autoclave.^129^ XPS analysis revealed that the amount of Ni^3+^ sites increased as the crystallinity decreased. This is significant because Ni^3+^ is considered a harder Lewis acid than Ni^2+^, and while both OH^−^ and Cl^−^ are hard Lewis bases, OH^−^ is harder than Cl^−^.^129,134^ As a result, it is clear why OH^−^ preferentially attaches to the borders and defects of abundant Ni^3+^ sites during the reaction.^129^ While this protective layer could result in superior OER activity. Theoretically, increasing electron density around the catalyst layer can hinder further OH^−^ adsorption and O_2_ gas evolution.^23,77^

In 1 M KOH and 0.5 M NaCl, the partially crystalline catalyst achieved current densities of 100 mA cm^−2^ and 500 mA cm^−2^ at overpotentials of 227 mV and 257 mV, respectively. In comparison, the highly crystalline anode required 34 mV and 97 mV greater overpotential under the same conditions.^129^ This is due to the smaller number of active sites on the highly crystalline NiFe-LDH, limiting the amount of OH^−^ adsorption, reducing the conversion rate of OH^−^ to O_2_ and why a more linear polarisation curve is seen from the partially crystalline NiFe-LDH. Stability tests were conducted over 24 hours at 100 mA cm^−2^ in an alkaline seawater solution; partially crystalline NiFe-LDH fluctuated by 20 mV, displaying good catalytic stability with a degradation rate of only 0.2 mV h^−1^. The partially crystalline electrode was more efficient with higher catalytic activity for the OER reaction.^129^

To further illustrate the HSAB theory, Guo *et al.* synthesised a hard Lewis acid layer (Cr_2_O_3_) onto an existing highly active OER electrocatalyst (CoO_*x*_) to create a bifunctional Cr_2_O_3_–CoO_*x*_ with no strong alkali buffers added to the electrolyte and only minimal filtration used for larger solids.^128^ The hard Lewis acid layer was added due to significant current attenuation (∼47%) of CoO_*x*_ in 1 M KOH & seawater after 100 h, which is attributed to the evolution of chlorine species and insoluble precipitation.^128^ Cr_2_O_3_ was synthesised as a selective layer *via* a thermal decomposition method and was selected due to Cr being the hardest Lewis acid amongst transition metals, with a p*K*_a_ value of 2.05. Titanium (Ti) follows closely at p*K*_a_ of ∼3.00. The Cr_2_O_3_–CoO_*x*_ electrocatalyst in natural seawater can achieve 150 mA cm^−2^ and 400 mA cm^−2^ at a cell voltage of 1.89 V and 1.99 V (Fig. 15b), respectively, with an *iR* compensation of 75%. Impressively, the cell can reach 1 A cm^−2^ at 1.87 V *via* increasing the operating temperature to 60 °C and remain stable at 500 mA cm^−2^ for 100 h. Applying a hard Lewis acid layer is a new technique that has served as a valuable method for improving the corrosion resistance to Cl^−^ ions. A further study could evaluate the catalytic performance in an alkaline environment.

Enhancing the catalytic activity of cutting-edge OER electrocatalysts remains a significant endeavour, but increasing the selectivity of distinct catalyst layers is gaining more prominence in the field. As such, some recent studies have solely focused on this aspect, aiming to address the challenge of improving the selectivity of OER catalysts. Bhardwaj *et al.* synthesised an ultra-thin inert silicon oxide layer (SiO_*x*_) on a Pt thin film electrode to show the effectiveness of SiO_*x*_ overlayers at repelling Cl^−^ ions (Fig. 15c) in a 100% ClOR region (>500 mV) in acidic and near neutral pH conditions.^130^ The SiO_*x*_/Pt was prepared using a photochemical method (electron-beam evaporation). A range of electrochemical tests were conducted to investigate the ability of the SiO_*x*_ to mitigate Cl-adsorption. The OER onset potential in Cl-free electrolytes is the same for bare Pt and SiO_*x*_/Pt electrodes at acidic pH. In a Cl^−^ electrolyte, the potential for the ClOR for the bare Pt is observed at (∼1.35 V) 270 mV lower than SiO_*x*_/Pt electrode (Fig. 15d). The saline electrolyte of 0.5 M potassium bisulphate (KHSO_4_) + 0.6 M potassium chloride (KCl) (set to mimic actual seawater conditions) (Fig. 15e), no oxidation peak related to the ClOR is observed at 1.35 V with the SiO_*x*_/Pt electrode, demonstrating the ability of the SiO_*x*_ to hinder the transfer of Cl^−^. Importantly, what the study highlights is that the OER selectivity of the SiO_*x*_ layer is impressive, given the unfavourable conditions for the OER, using a Pt, notably a poor OER electrocatalyst and using a higher Cl^−^ concentration (0.6 M) than other studies^23,51,82,94–96,107,120,129^ as well as using an acidic environment.^130^ Furthermore, utilising SiO_*x*_ overlayers on more catalytically active OER electrocatalysts is anticipated to yield substantial enhancements in OER faradaic efficiencies within the same potential range investigated in this study. This is particularly relevant given Pt's observed minimal OER partial current densities in the aforementioned potential range.^130^

In 2022, Yan *et al.* synthesised a MnO_2_ on a CC to solely investigate the OER selectivity of the MnO_2_ nanosheet arrays.^131^ MnO_2_/CC was prepared using a facile hydrothermal method, and implementation of transition metals (Fe, Co and Ni) was subsequently achieved *via* an immersion step in a salt solution. The study aimed to investigate the OER selectivity of MnO_2_ in pure unbuffered seawater. A constant current of 100 mA cm^−2^ was applied for 30 minutes, and a following electrolyte titration revealed the amount of hypochlorous acid formed over the test. The selectivity of the MnO_2_/CC electrode was 66.7%; it was found that increasing the content of Mn^4+^ enhanced the adsorption of OH^−^ ions, thus increasing the OER selectivity.^131^ As stated previously, performance was not the focus of this study, as MnO_2_/CC required 1098 mV to achieve 100 mA cm^−2^ in seawater. Interestingly, doping transition metals Co, Fe, and Ni separately had no positive impact on performance and resulted in increased overpotentials of 1140 mV, 1236 mV and 1465 mV, respectively, at 100 mA cm^−2^. This can be attributed to the reduced Mn^4+^ content when doping transition metals into MnO_2_/CC. Overall, the study proves MnO_2_/CC has sufficient tolerance to ClOR in seawater electrolysis and thus suggests valuable insight for a catalyst layer for further investigation with more active OER electrocatalysts.

### 4.5. Electrode design and application in DSWE

Thus far, the main focus of our review has been the development of OER catalyst materials that would allow for the direct electrolysis of seawater using the current water electrolyser cell configurations. However, to aid the commercialisation of the technology, the focus needs to encompass the entire electrolyser rather than just catalysts. It is worth highlighting that recent innovative efforts have been aimed at modifying the cell configuration to meet the unique requirements of seawater electrolysis. Dresp *et al.*, in 2020, implemented an asymmetric chamber design within an AEM that holds two different electrolytes, 0.5 M KOH at the anode and 0.5 M NaCl at the cathode, mitigating ClOR at the anode and allows the cell to operate with similar performance to using fresh water.^135^ In 2022, Xie *et al.* present a vastly novel cell modification that uses an *in situ* water purification step; this is achieved using a hydrophobic PTFE-based waterproof breathable membrane as a gas–path interface while using concentrated KOH as a self-dampening electrolyte (SDE), which allows the cell to run for 3200 h at 250 mA cm^−2^.^136^ However, we acknowledge that neither of these configurations supports the need for complex corrosion-resistant anodes. Since incumbent PEM and AEM cells require a membrane for ion conduction and ensuring optimal safety of separating gaseous products H_2_ and O_2_. The membrane presents a further challenge to seawater electrolysis, specifically unwanted ion crossover and biofouling of the membrane. With this in mind, we explore a few studies on membraneless electrolysers and, as a result, their application to a seawater electrolyte, allowing the electrocatalysts analysed in this review to be relevant.

**Fig. 16:**
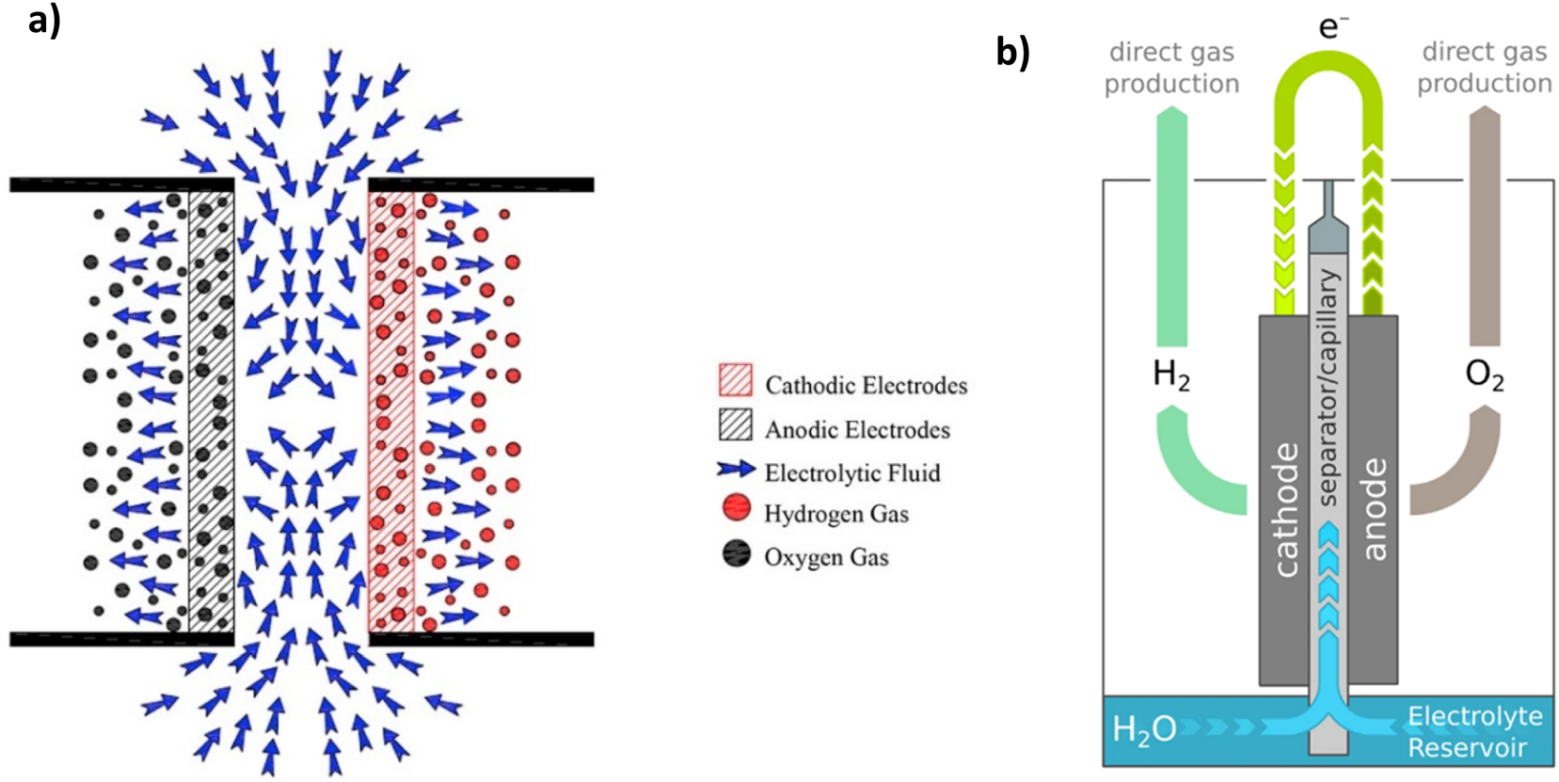
(a) Membraneless ‘flow-through’ DEFT electrolysis operation, reproduced from ref. 137 with permission from Elsevier, copyright 2015. (b) Capillary-fed electrolysis cell, reproduced from ref. 139 under Creative Commons License (CC BY).

The major issue with membraneless electrolysers is the gas separation, the most widely reported types of membraneless electrolysers are ‘flow-by’ or ‘flow-through’ (Fig. 16a) type, where gas separation is achieved through electrolyte flow along parallel electrodes. The most successful flow-through electrolyser was introduced by Gillespie *et al.* in 2015.^137^ The flow-through membraneless electrolyser was the first of its kind and utilised parallel nickel mesh electrodes and was named the divergent electrode-flow-through (DEFT) (Fig. 16a). The electrolyser could impressively reach current densities of up to ∼3.9 A cm^−2^ at 3.5 V, using a 6.9 M KOH electrolyte at a flow rate of 0.2 m s^−1^, producing H_2_ with a purity of 99.83%.^137^ Remarkably, the membraneless electrolyser was on a large scale (1 kW), making it the largest installation in the peer-reviewed literature.^137,138^ The study highlights the benefit of coating the electrodes. It suggests that using catalysts that are selective for OER and HER and increasing the operating temperature will result in much greater performance and increase the development of compact alkaline electrolysers for various applications such as DSWE. As a result, using selective anodes discussed in Section 4.4.3 could be a worthwhile investigation for future research.

However, ‘flow-by’ or ‘flow-through’ type membraneless electrolysers experience non-negotiable gas crossover.^138^ Thus, some of the latest research is looking at capillary gas flow electrolysers, which are an example of a quasi-membraneless electrolyser, since gas diffusion electrodes are coupled with membranes. Hodges *et al.* present a state-of-the-art example of a capillary gas flow through an electrolyser that could be used in a seawater environment.^139^ A thin layer of 27 wt% KOH is continuously fed to the NiFeOOH anode and Pt/C cathode *via* spontaneous capillary action (Fig. 16b). A porous, hydrophilic separator is marginally submerged in an electrolyte, generating a capillary-induced upward in-plane movement of the electrolyte. The electrodes draw in a thin layer of electrolyte laterally from the separator, and any H_2_ or O_2_ gases formed transport within the electrolyte, creating bubble-free electrolysis.^139^ This means that the cell is not disadvantaged by bubbles blocking the electrodes, allowing full use of the active sites. Remarkably, the cell can achieve 500 mA cm^−2^, needing only 1.51 V at 85 °C, resulting in an energy consumption of 40 kW h per kg H_2_ (98% energy efficiency). The cell provides valuable insight into the simplified balance of plant and efficient energy consumption. Furthermore, coupling this cell with an ion-selective or (poly) anion-doped anode, as explored in this review, could be used effectively in a seawater environment.

## 5. Summary and outlook

Seawater electrolysis shows excellent promise and presents a genuine opportunity for an inexhaustible source of green hydrogen and an effective method for inexpensive energy storage. As reviewed, there has been significant improvement in the development of OER electrocatalysts for alkaline seawater electrolysis, with a growing trend towards modified or tuned earth-abundant catalysts. The challenging task of improving sluggish OER reaction kinetics while mitigating chloride corrosion has been studied, and promising research has been highlighted. NiFe LDHs have shown the highest activity due to their significant electrochemical active surface area and ability to retain the interlayer spaces, effectively accommodating a diverse range of anionic species, as discussed in detail in this review. While adding precious metals and metals of high economic value generally aids in the performance of earth-abundant electrocatalysts, the majority of the literature is now focused on reducing the precious metal loadings. Remarkably, the performance of pure earth-abundant electrocatalysts is nearly comparable to those with precious metals. This highlights that additional cost does not necessarily lead to a significant boost in performance. However, the critical addition of Mo to electrocatalysts - notably when S-doped - demonstrates impressive and consistent performance in alkaline seawater electrolytes, as observed with Mo–Ni_3_S_2_/NF for over 500 hours at 100 mA cm^−2^. It is incredibly challenging and time-consuming to synthesise electrocatalysts that contain metals with high economic value. Several studies have shown that at least a two or three-step synthesis is needed, while a single hydrothermal synthesis only takes a few hours. Adding an extra step could extend the time required to several hours or even a day, which limits the ability to scale up the catalyst. The hydrothermal method is a consistently observed synthesis method used thus far, as it is a helpful method to control the surface chemistry, particle morphology, and grain size of a catalyst.^140^

Alkaline seawater electrolysis requires stability and durability, which has been a primary concern. Upon review, researchers have found that the electrostatic repulsion strategy is a widely explored approach to repel chloride ions, resulting in increased durability for OER electrocatalysts without interfering with active sites on the catalyst's outer layer. However, it is apparent that only a few studies mention that increasing electron density around the catalyst layer hinders further OH^−^ adsorption and thus O_2_ gas evolution, meaning a high activation energy is required to overcome the O–O coupling thermodynamic barrier.^23,77^ It's noteworthy to see lab-scale studies reaching over 1000 hours of testing,^23,80^ but for practical commercial applications, these catalysts must undergo extended testing to mimic realistic use.

In short, a suitable OER electrocatalyst for DSWE should be robust, be able to repel Cl^−^ ions effectively, have high electrocatalytic activity and have a high electrochemical surface area that is simple to synthesise while being composed of earth-abundant metals/materials. Numerous studies suggest significant strides have been made in the research field, yet the successful commercialisation of DSWE remains a few years away. To make meaningful progress, a fusion of pioneering catalyst advancements and innovative engineering designs is necessary to enhance electrocatalytic performance further.

## Conflicts of interest

The authors declare no conflict of interest.
